# Assessing Functional Capacity in Myalgic Encephalopathy/Chronic Fatigue Syndrome: A Patient-Informed Questionnaire

**DOI:** 10.3390/jcm13123486

**Published:** 2024-06-14

**Authors:** Kristian Sommerfelt, Trude Schei, Katharine A. Seton, Simon R. Carding

**Affiliations:** 1 Children and Youth Clinic, Institute of Clinical Medicine 2, University of Bergen, P.O. Box 7804, 5020 Bergen, Norway; 2 Norwegian ME Association, Nedre Slottsgate 4 M, 0157 Oslo, Norway; 3 Quadram Institute Biosciences, Norwich Research Park, Norwich NR4 7UQ, UK; katharine.seton@quadram.ac.uk (K.A.S.); 4 Norwich Medical School, University East Anglia, Norwich NR4 7TJ, UK

**Keywords:** ME/CFS, chronic fatigue syndrome, functional capacity, post-exertional malaise, questionnaire, survey

## Abstract

**Background:** Myalgic encephalomyelitis/chronic fatigue syndrome (ME/CFS) is an acquired disease with significant morbidity that affects both children and adults. Post-exertional malaise is a cardinal symptom of ME/CFS and impacts a patient’s functional capacity (FC). The absence of effective tools to assess FC has significant consequences for timely diagnosis, clinical follow-up, assessments for patient disability benefits, and research studies. In interventional studies, the inability to assess FC can result in an incomplete assessment of the potential benefit of the intervention, leading to beneficial treatment outcomes being missed. **Methods:** Using extensive, repeated patient feedback, we have developed a new questionnaire, FUNCAP, to accurately assess FC in ME/CFS patients. The questionnaire consists of eight domains divided by activity types: A. personal hygiene/basic functions, B. walking/movement, C. being upright, D. activities in the home, E. communication, F. activities outside the home, G. reactions to light and sound, and H. concentration. **Results:** Through five rounds of anonymous web-based surveys and a further test–retest validation round, two versions of the questionnaire were developed: a longer version comprising 55 questions (FUNCAP55), developed for improved diagnostic and disability benefit/insurance FC assessments; and a shorter version (FUNCAP27) for clinical patient follow-up and potential use in research. Good reliability and validity and negligible floor and ceiling effects were found, with comparable findings in all aspects in both a large Norwegian (*n* = 1263) and a separate English-language international sample (*n* = 1387) demonstrating the validity and reliability of FUNCAP. **Conclusions:** Our findings support the utility of FUNCAP as an effective, reliable and valid tool for assessing FC in ME/CFS patients.

## 1. Introduction

Myalgic encephalomyelitis/chronic fatigue syndrome (ME/CFS) is an acquired disease affecting children and adults with an estimated prevalence in the US, when post-exertional malaise (PEM) is present, of 0.8% [1]. In addition to PEM, central ME/CFS features are new onset of severe reduction in pre-illness FC for all activities, extensive fatigue, cognitive symptoms such as “brain fog”, non-restorative sleep, pain, orthostatic intolerance, and sensory hypersensitivity [2]. Extensive reviews by committees in the US and UK concluded that ME/CFS is a serious, systemic disease with multiple symptoms of new onset and no known cause or effective treatments [3,4].

For diagnosis, the Norwegian National Guidelines for ME/CFS recommend using the Canadian Consensus Criteria [5] and Fukuda criteria [6] for adults and the Jason criteria [7] for children and adolescents below 18 years of age [8]. The presence of PEM is mandatory in these, except for the Fukuda criteria, where it is an optional symptom [6]. These guidelines state that PEM is a cardinal ME/CFS symptom, making its presence probable in Norwegian patients diagnosed with ME/CFS by specialists. PEM is also reported in long COVID-19 patients [9]. Although there is considerable overlap between long COVID-19 and ME/CFS [10], with some long COVID-19 patients receiving an ME/CFS diagnosis [9], the PEM experienced by long COVID-19 patients has distinct differences to that experienced by ME/CFS patients [9].

A review of patient-reported outcome measures (PROMs) used in ME/CFS studies found that few assessed physical and cognitive FC [11]; those that did had poor validity and reliability. Existing assessment tools commonly inquire about patients’ abilities to perform specific activities without considering the potential consequences on PEM. This approach may lead to varied patient interpretations and responses. Some may indicate they can perform an activity, overlooking the PEM it induces, while others may report an inability to perform the same activity due to PEM. In a 2015 systematic review of PROMs assessing activity limitations and participation restrictions in individuals with more widely defined chronic fatigue syndrome, typically lacking the mandatory presence of PEM [12], SF-36 [13] was the most frequently used tool [12]. The review found SF-36 scales to have unknown internal consistency, content validity, and interpretability when used in this setting. It considered the CFS-Activities and Participation Questionnaire (CFS-APQ) [14] to be the best available tool for assessing activity levels in this setting. However, although the internal consistency of the CFS-APQ was good [15], its test–retest reliability was inadequately assessed, and its content and construct validity was moderate [12]. Another limitation of the CFS-APQ is the absence of incorporating PEM as a consequence of undertaking activities into the questionnaire items and response scales. Other than CFS-APQ, we have found no other questionnaire specifically targeting FC in ME/CFS, highlighting the need to develop tools to accurately assess and record FC in ME/CFS.

The accurate evaluation of FC in ME/CFS patients is paramount for several reasons. First, a more than 50% reduction in FC is typically required for receiving an ME/CFS diagnosis in Norway [16]. Second, broadly assessing FC reduction in ME/CFS is essential in health insurance evaluation and when health authorities assess the right to, and need for, disability benefits [17]. Third, in medical follow-up consultations of ME/CFS patients, accurate knowledge of FC is vital for appropriate patient care. Fourth, in ME/CFS interventional research, accurate assessment of changes in FC after an intervention is essential. In this setting, assessing the severity of symptoms such as fatigue, pain, nausea, and sleep disturbance, whilst still important, is inadequate. If an intervention has a positive effect and reduces symptoms, patients may, consciously or unconsciously, increase their total level of activity and reach a pre-intervention symptom burden, but with increased FC as a benefit. Thus, assessing symptom severity only may miss a positive effect of the intervention. Fifth, when evaluating a novel potential biomarker for ME/CFS, assessing biomarker correlation with FC severity will strengthen evidence for its validity.

The primary aim of the present study was to develop a questionnaire demonstrating the range and extent of FC capacity and FC reduction in ME/CFS patients. To achieve this, the questionnaire needs to be accurate and comprehensible for healthcare workers and other personnel for use in clinical work and in disability benefit/insurance assessments. The secondary aim was to develop a shorter version of the resultant questionnaire suitable for assessing ME/CFS FC in follow-up, clinical care, and research studies.

## 2. Methods

SPSS v28 was used for all statistical analyses. The study methods and protocol were reviewed by the Regional Ethics Committee for Research at Haukeland University Hospital, Bergen, Norway, with application number 746956. It concluded that as the respondents were anonymous, ethical review was not required.

### 2.1. Questionnaire Development

Anonymous, internet-based surveys were the basis for developing the questionnaire. In all, six survey rounds were undertaken, with the initial three Norwegian rounds targeting the questionnaire’s content. The fourth round was undertaken internationally in English to assess the questionnaire’s validity by comparing the findings in another independent cohort of patients. Similar findings to those in the Norwegian cohort would support the questionnaire’s validity and generalizability. The fifth round was the main Norwegian survey, and the sixth round was a Norwegian test–retest round incorporating two surveys. Carers could answer on behalf of patients who were too ill to answer themselves. Invitations to participate in the surveys were issued through social media accounts belonging to the Norwegian ME Association (NMEF). The NMEF offers a range of moderated support groups on Facebook for both patients and carers, with approximately 11,000 participants and with some overlap between groups. Approximately half of these participants were non-members of the NMEF. The NMEF also has followers on Facebook, Twitter (X), and Instagram, supporting a reach far beyond NMEF membership.

In line with the recommendations of reviews on PROM development in general [18] and specifically for ME/CFS [11], a strategy of item identification and questionnaire development relying on ME/CFS patient feedback was employed. This was prioritized because of the inherent “hidden” nature of delayed PEM [19,20,21,22]. Healthy controls (HCs) were included in the third and fifth (both Norwegian) survey rounds, but not in item development, as it was not our aim to address their range of FC. Also, whilst HCs were included to demonstrate their response to the items, they were not included in the internal consistency or test–retest validation analyses which were carried out on data from Rounds 4 and 5. HCs were healthy family members or other relatives or friends of ME/CFS patients, with their HC status confirmed using the tick box “I do not have ME/CFS or other disability”.

The removal, modification, and, in some instances, addition of items in consecutive survey rounds were based on invited, critical feedback from survey respondents, moderated by K.S. and T.S. No formal text analyses were performed (see Supplementary Material File S1).

We assumed that the Norwegian Nnational Guidelines for ME/CFS diagnosis [8] were generally adhered to, making the presence of PEM in respondents likely. To classify disease severity, we used the International Consensus Criteria (ICC) definitions: *very severe*: totally bedridden and in need of help with basic functions; *severe:* mostly bedridden; *moderate:* mostly housebound; *mild:* at least a 50% reduction in activity level compared to before disease onset. In addition, we added the category *better than mild:* symptomatic, but less than a 50% reduction in activity level [16].

The SurveyMonkey platform was used in all survey rounds. Only one response per IP address was allowed in all survey rounds for the main ME/CFS respondent. Using this mode in SurveyMonkey had the major advantage that respondents could answer in repeated sessions as long as the survey periods remained open. Encouraging the recruitment of HC respondents in Rounds 3 and 5 was enabled by adding a secondary survey (identical content). Based on critique from respondents in Round 3, the secondary survey was based on the option allowing for more than one respondent in SurveyMonkey, as more than one HC could potentially be recruited from the same household/IP address. This option did not have the option of being left open over time until completed but had to be answered in one go. In Round 5, we also stated that if there were more than one ME/CFS patient within a household, they could also use this secondary survey.

The length of time for which the surveys remained open was decided separately for each round to allow for the initial fast response frequency to fall and to enable more severely affected respondents to participate, thereby increasing the likelihood of more representative sampling. The exception was Round 1, which was planned to be kept open only for the time needed to achieve at least approximately 200 respondents, allowing us to obtain initial feedback to formulate the initial questionnaire. For subsequent initial rounds, the surveys were left open for three to four weeks. The first three rounds included a question at the end of the survey which invited respondents to provide open-ended criticism or ideas for the authors to consider and incorporate in the development of FUNCAP.

In all questionnaire rounds, respondents were encouraged to complete them with someone who regularly sees or visits them. The reason for this was that cognitive fatigue (“brain fog”) and memory issues are common symptoms of ME/CFS [3,4]. Additionally, before Round 1, when piloting the questionnaire in clinical settings with a patient and one or more close person(s) giving feedback, it became clear—especially for the more severely affected patients—that conferring between the patient and close person improved precision. Instructions, specific items, and response scales are described in Supplementary Material File S1.

### 2.2. Initial Survey Rounds Developing FUNCAP Questionnaire Items

Round 1. Items were sourced primarily from published PROM questionnaires (Supplementary Material File S1), *including* the CFS-APQ [14] and a validated generic Norwegian 40-item questionnaire (hereafter called the NFS) [23] targeting FC based on the WHO ICF 2001 framework [24,25]. The NFS was developed using a patient focus group (*n =* 386) and included factor analysis [23]. Items relevant to the most severely affected ME/CFS patients were sourced from the Activities of Daily Living Score (ADLS) and the Tolerance Score for Sensory Stimuli (TSSS), used in a previous study targeting *severe* and *very severe* ME/CFS [26]. In many cases, items from these sources were modified. Examples are improving the precision of item wording and introducing or changing item specifications such as distances and time periods (see Supplementary Material File S1).

A total of 36 items was used in Round 1, 9 of which targeted PEM (not included after Round 1). Five of the 36 items were created by the authors (K.S. and T.S.) based on their extensive ME/CFS clinical experience, communication with national and international colleagues working with ME/CFS, and patient contact (Supplementary Material File S1). The remaining 31 items were sourced from the NFS (14), CFS-APQ alone (2), ADLS alone (7), TSSS alone (2), and from the NFS and CFS-APQ (6) (see Supplementary Material File S1). In each of these 36 items, we asked: “How often during the last week have you undertaken these activities?”. The response options were *never, rarely, sometimes, often, very often*. Nine additional items targeted PEM and were written by K.S. and T.S., asking: “How often during the last week have you experienced the following?”, scored for frequency as above (see Supplementary Material File S1).

Groups of items were ordered in domains according to activity type. This was partly to facilitate respondents answering the questionnaire by moving through logical sections. This was also in line with our conceptual model (this study’s primary aim) of developing a tool that demonstrated FC across important activity domains for clinical work and other use [5,14,26].

The domains were as follows: (A) personal hygiene/basic functions, (B) walking/movement, (C) being upright, (D) activities in the home, (E) communication, (F) activities outside the home, (G) reactions to light and sound, and (H) concentration. These were partly chosen on the authors’ clinical experience, but mainly based on previous research. The sources for these domains were as follows: for A (personal hygiene/basic function), ADLS and NFS; for B (walking/movement), CFA-APQ and NFS; for C (being upright), CFS-APQ; for D (activities in the home), CFS-APQ and NFS; for E (communication), CFS-APQ and NFS; for F (activities outside of the home), CFS-APQ; for G (reactions to light and sound), TSSS; for H (concentration), NFS (see Supplementary Material File S1). Some changes were made to the domains following respondent feedback (described in Supplementary Material File S1 and in the Results section). Demographic data were collected in the study rounds.

Round 1 was undertaken from the 11–15 March 2022 (five days), yielding 290 respondents who completed the survey. A frequent critique from respondents was that activity frequency did not address the consequences of undertaking the activity, including their consequences on the capacity to perform other activities. Many respondents described choosing not to undertake various activities because doing so repeatedly resulted in negative consequences, including an extended, decreased capacity for all activities and increased symptoms (PEM). Many noted that the frequency of undertaking specific activities depended on choice, preferences, and access to support from others. Instead, respondents suggested asking questions about the consequences of undertaking given activities. Also, more precise descriptions of items were requested, including distance and time, replacing terms such as “a short” or “long walk” with “Walk 100 m to 1 km”. Many commented that not enough items were appropriate for the least and most severely affected. Also, not enough items adequately covered the negative consequences of being in an upright position (sitting or standing) and hypersensitivity to light and sound on FC.

Round 2. A major revision to Round 1 in the Round 2 version was introducing a six-point ordinal response scale targeting the consequences of undertaking an activity on the capacity to carry out other activities, and the duration of such consequences: *1: I cannot do this activity without severe deterioration. 2: Very much—can do nothing else the same day or days after. 3: A lot—can do nothing else the same day. 4: Fairly much—reduce activity level the same day/have to take extra rest before or afterwards. 5: Some—but rarely affects other activities. 6: Unproblematic—does not affect other activities.* A new domain was also introduced, namely *being upright*, with relevant items moved to this domain and expanded on. More items targeting sensory exposure (light and sound) were introduced, including two written by K.S. and T.S. Specific time and distance criteria were introduced to items in several domains, most of them based on Round 1 feedback. The wording was changed in several items, partly based on respondent feedback. In the resulting 54-item Round 2 questionnaire, 6 of the 54 items were new, written by K.S. and T.S. (see Supplementary Material File S1).

After Round 2, the number of domains increased from seven to eight according to activity types.

Round 2 data were collected from 22 April to 19 May 2022 (27 days) and yielded 435 respondents answering all FUNCAP items. Many missed specifications and responses to questions regarding what type of day they were having (good/bad/average?). A frequent response was that there were activities they were unable to perform with no corresponding scoring option. Specifying showering as either standing or seated was requested. Suggestions were made to separate “*Gotten dressed in regular clothes*” and “*got out of bed*” into separate items. Several respondents commented that the item regarding reading did not specify the type of text. There was a repeated critique of items with numeric descriptions such as “… more than 10 min”, suggesting “for 10 min” instead. Another example was requests for specifying the length of time spent as a passenger in a car. Several still stated the need to further clarify the degree to which the sum of chosen activities resulted in PEM.

The majority of Round 2 ME/CFS respondents (75%) reported that they felt the questionnaire was highly suitable for describing their FC.

Round 3. As described for Round 2, revisions were implemented, resulting in Round 3. We added a response option of being unable to undertake an activity at all. Respondents were asked to answer by considering a typical day. We specified “Reading and understanding a non-fiction text/official document (at least one A4 page long)”. Several other adjustments, mainly in terms of wording, were made (see Supplementary Material File S1).

Round 3 was undertaken from the 30 May to the 21 June 2022 (23 days), yielding 536 ME/CFS respondents that answered all FUNCAP items. Some non-substantial suggestions for changes in wording were made (see Supplementary Material File S1).

The FUNCAP questionnaire version used in Round 3 was used extensively in clinical practice by K.S. and by several other clinicians with extensive knowledge and experience in diagnosing and managing ME/CFS patients of all ages and severity degrees. Feedback from these clinicians resulted in minor adjustments to the wording of some questionnaire items. It was deemed most useful and effective in clinical settings when given in person or e-mailed to patients for completion in advance of consultation. The final FUNCAP questionnaire consisted of 55 items spanning eight domains and is herein referred to as FUNCAP55. We also asked if respondents could recruit respondents without ME/CFS, who could be family, relatives, friends, or others, as HCs.

Round 4. An English version was developed to provide an English-language version of FUNCAP that was not only a translation, but also a separate survey. An additional motivation was the potential to contribute to the evaluation of FUNCAP’s validity and reliability by comparing the results to the Norwegian version. The FUNCAP55 questionnaire from Round 3 was revised, with minor changes to the wording of some items and descriptions in the response scale (see Supplementary Material File S1). Regarding general item responses, respondents were asked to “*Choose the option that is closest to your experiences*”. The questionnaire was then translated into English by this study’s authors, K.S. and T.S. (see Supplementary Material File S2). Invitations to participate were shared openly on Twitter and were retweeted by several ME patient organizations. Invitations were also shared in international, English-language, ME/CFS patient groups on Facebook, and through the Open Medicine Foundation’s newsletter.

Round 5. The Round 5 FUNCAP55 questionnaire was very similar to Round 4, with two items (visiting and receiving a visit from a friend) substituted with the item “Participating in a conversation with three people for approximately ½ h”. Also, some wording in the response scale was adjusted. Respondents were asked to “Base their response on a typical day during the last month—not the worst nor the best”. The questionnaire was then translated into English by study authors K.S. and T.S. and back-translated to Norwegian by an experienced psychologist proficient in the English language and with extensive knowledge of questionnaire development and of ME/CFS. The back-translated version 5 was virtually identical to the original Norwegian version 5, although some minor wording changes were made to the response scale (see Supplementary Material File S1).

After the last FUNCAP item, respondents were asked, “How did you experience answering FUNCAP? To what extent do you agree with following statements: 1. Strongly agree. 2. Agree. 3. Disagree. 4. Strongly disagree”. The questions were “Easy to understand? Easy to know what to answer to the questions. Gave a correct view of my illness-situation. Needed help from others to answer. Needed several breaks when filling out”.

They were also asked how well they felt the questionnaire assessed their FC in four general areas on the following scale: 1: *Very bad*, 2: *Bad*, 3: *Good*, 4: *Very good*. The areas were *sensitivity to light and sound*, *being upright*, *physical function, and cognitive function*.

The demographic data collected included year of birth, age at ME/CFS onset, gender of the patient completing the survey (or person answering on behalf of ME/CFS respondent if too sick to answer), and who made the initial ME/CFS diagnosis. Only respondents completing FUNCAP questionnaire items were included in this study. Missing data on background variables were not used to exclude respondents. SPSS versions 27 and 28 were used for all statistical analyses. Two-tailed *p*-values were adhered to in all analyses.

We also relied on ME/CFS respondents to recruit respondents without ME/CFS, including family, relatives, friends, or others, as healthy controls (HCs).

### 2.3. Strategy for Shortened Questionnaire Version

Having arrived at the final extensive questionnaire targeting the primary study aim, a shortened version for clinical follow-up and potential research use was developed to address this study’s second aim. Limiting the of number of items to improve response accuracy is important given the presence of “brain fog” in ME/CFS respondents. We calculated the correlation between each individual item score within a domain and the corresponding mean item score for that domain. Items with correlations at or above 0.7 were chosen as candidate items to retain whilst maintaining domain internal consistency in line with the Classical Test Theory [27,28]. However, incorporating the evaluation of face-value item content is also important when selecting items to retain so as not to automatically retain those with the highest correlation between item score and mean domain item score [27,28]. This is vital to minimize the reduction in questionnaire construct validity from a long to a short form [27,28]. In this study, this entailed taking into consideration that the retained items should have content relevance across the range of ME/CFS severity degrees. To aid in identifying optimal items to retain, we assessed the mean score and SD for all items stratified by ME/CFS severity degree, including severe and very severe. We strived to, as often as possible, retain at least one item in each domain with mean scores between 2 and 4 (i.e., near the middle range of the item score) across all severity degrees. These strategies should also contribute to minimizing floor and ceiling (F/C) effects. Deciding on the final number of questionnaire items was guided by a strategy of ending up with approximately half the number of items.

### 2.4. Statistical Evaluation of Questionnaire Sub-Scores

Once the FUNCAP questionnaire items were developed and finalized, we described the statistical characteristics of the questionnaire sub-scores and analyzed the reliability and validity of the FUNCAP questionnaire. We used Rounds 4, 5, and an additional Round 6 (test–retest) for these analyses and included data from respondents under the age of 60, as ME/CFS phenotype differs with older age [29]. This was also aimed at reducing the potential effect of increased frequency of unrelated fatiguing conditions with increasing age.

#### 2.4.1. Descriptive Statistics

For each FUNCAP sub-score, we described the mean value, standard deviation, range, and percentage with the highest and lowest possible score. Floor and ceiling (F/C) effects were defined as the proportion of respondents scoring the highest possible score (ceiling, i.e., 6) or lowest possible score (floor, i.e., 0) across the eight A to H domains. F/C effects were classified as significant if ≥15%, moderate if 10% to <15%, minor if 5% to <10%, and negligible if <5% [30,31].

#### 2.4.2. Reliability

##### Internal Consistency

The internal consistency of questionnaire domains A to H in the final version (Round 5) was assessed using Cronbach’s alpha coefficient analyses for each domain.

##### Round 6: Test–Retest Reliability

Reliability was further assessed in a separate test–retest survey round (Round 6) where the same respondents answered the questionnaire two weeks apart. The shortened version of the questionnaire (described below in Section 3.4.1) was used for this assessment. Only responses from ME/CFS respondents were used, since the assessment of FC was not valid for HCs (see above). The ME/CFS respondents were also asked how well they felt the questionnaire assessed their FC in various domains on the following scale: 1: very bad, 2: bad, 3: good, 4: very good. To assess reliability, the Intraclass Correlation Coefficients (IcCCs) with 95% confidence intervals were calculated for the A to H domain scores and total scores using a 2-way mixed model with absolute agreement. The interpretations were as follows: excellent IcCC reliability: IcCC > 0.90; good: 0.75–0.90; moderate: 0.5–0.75; poor: <0.5 [27].

#### 2.4.3. Validation

Our main overall validation strategy was the “known group” validation method. This entails testing hypotheses about what results we would expect to obtain if the FUNCAP was a valid measure of reduced FC among ME/CFS respondents. We would then expect (1) higher FUNCAP sub-scores for HC respondents compared to those for ME/CFS; and (2) a monotonic relationship between sub-scores at different levels of ME/CFS severity such that mean scores at each successive level of severity were lower than the prior level, indicating a decline in FC with increasing level of MR/CFS severity.

Content validity was explored by assessing to what extent the final set of FUNCAP items reflected functions in the WHO ICF 2001 framework [24].

A separate, international, English-language survey was used to further assess FUNCAP’s validity.

## 3. Results

### 3.1. Respondent Demographic Information

The demographic and ME/CFS severity data gathered in all rounds are described in Table 1. Female respondents constituted around 90% vs. 10% males across all survey rounds (Table 1). ME/CFS severity was comparable across all survey rounds, including the English/international Round 4 (Table 1).

### 3.2. Initial Survey Rounds

The results from the initial survey Rounds 1 to 3 are described in Section 2.2 and in Supplementary Material File S2.

### 3.3. Final Survey Rounds

#### 3.3.1. Round 4—English Version

Round 4 was undertaken between 9 September and 17 December 2022 (100 days) and yielded 2128 initial respondents, of whom 1945 had been diagnosed with ME/CFS and answered all FUNCAP items. Of these, 1387 (71%) were below 60 years of age and were included in the analysis (Table 1 and Table 2). There were 334 (24%) respondents from the UK, 288 (21%) from the USA, 139 (10%) from Australia, 108 (8%) from Canada, 70 (5%) from Northern Ireland, 68 from Norway (5%), 67 (5%) from Sweden, 60 (4%) from Germany, 177 (13%) from other European countries, and 66 (5%) from outside Europe. The majority of the respondents classified their disease severity as moderate (Table 2).

#### 3.3.2. Main Round 5 Respondent Characteristics

Round 5 was open from 6 March 2023 to 6 April 2023 (31 days) and yielded the main data used in the present study. It used the final FUNCAP55 questionnaire (see Supplementary Material File S2) with minor changes from the Round 4 version (see Supplementary Material File S1 for details). It included both ME/CFS and HC respondents. There were 1463 ME/CFS respondents who answered all FUNCAP items. Of these, 1263 (86%) were below 60 years of age and were included in the analysis (Table 2). Among the 1263 respondents, 1136 were from the survey with only one response allowed per IP address, and 127 were from the second Round 5 survey, in which more than one response per IP address was allowed. For 72 (6%) respondents, the questionnaire was completed by another person due to the patients being too ill to complete it themselves. In this last group, by mistake, we did not ask whether a consultant or general practitioner had diagnosed ME/CFS. Among the 223 HC respondents completing the FUNCAP55, 188 were below 60 years of age. Among these, 107 came from the second survey allowing for more than one response per IP address and 81 came from the first survey. Of the 188 HC respondents, 10 were excluded as outliers based on their scores on certain items being incompatible with their status as HCs.

Among the 1263 ME/CFS respondents included in the analysis, 878 (69%) were diagnosed in a hospital by a consultant and 313 (25%) by a general practitioner. For 72 (6%), we do not know whether the ME/CFS diagnosis was given by a consultant or a general practitioner. The questionnaire design precluded obtaining this information. Of the 72 respondents in this group, 9 respondents had *very severe*, 24 *severe*, 32 *moderate*, and 7 *mild* ME/CFS. Those with severe and very severe disease were included because they were most likely generally too ill to answer. Those with less severe ME/CFS were also included as they could have other reasons, such as having an intercurrent infection or other transitory reason, preventing them from answering. The most common severity degree was moderate (Table 2).

### 3.4. Round 5 Item Scores

#### 3.4.1. Creating a Shortened Version of FUNCAP55 (FUNCAP27)

Using the method described previously (Section 2.3), we created a shorter version of the FUNCAP55 based on Round 5 data. Pearson correlations between mean domain sub-scores and individual FUNCAP55 item scores in that domain were within the following ranges: A: 0.7 to 0.91; B: 0.68 to 0.93; C: 0.78 to 0.91; D: 0.82 to 0.90; E: 0.82 to 0.90; F: 0.70 to 0.90; G: 0.68 to 0.85; H: 0.56 to 0.81. Applying the method (Section 2.4) led to the 27-item FUNCAP27 (see Supplementary Material File S2). When stratified according to ME/CFS severity groups (*very severe* and *severe* were grouped together), most FUNCAP27 domains contained at least one item with a mean item score in the middle range (i.e., between 2 and 4) for every severity group. The exceptions were as follows: *very severe/severe*, where both domain D items had mean values < 1; *mild* in domains A and C, where all items had mean values above 4; *better than mild,* where all items across domains except domains F and H contained only items with mean values above 4. The latter group was not an intended target group, and the corresponding findings were not taken into account.

#### 3.4.2. FUNCAP Item Score Statistics

Next, we compared FUNCAP55 item scores reported by ME/CFS patients versus HCs to assess the magnitude of FC reduction among ME/CFS respondents. They had lower mean scores on all individual item scores compared to HCs (Table 3).

The difference in mean item scores (0 to 6) between ME/CFS and HC respondents on the FUNCAP27 ranged from 0.6 vs. 5.5 for item H27, “Managing a full working day (non-physical work such as office work, classes or lectures)”, to 5.4 vs. 6.0 for item H22, “Reading a short text, such as a mobile phone text message” (Table 3).

#### 3.4.3. Round 5 FUNCAP55 Respondent Evaluation

Respondents reported that the FUNCAP55 was easy to understand and gave an accurate picture of their illness (Table 4).

The following scale was used for ME/CFS respondents in Round 5 to evaluate how they felt the questionnaire assessed their FC in the individual activity domains: 1: *very bad*, 2: *bad*, 3: *good*, 4: *very good*. The results were as follows: distribution of responses for *sensitivity to light and sound*: 1: 1%, 2: 9%, 3: 69%, 4: 22%; distribution of responses for *being upright:* 1: 0.3%, 2: 11%, 3: 63%, 4: 26%; distribution of responses for *physical function:* 1: 1%, 2: 14%, 3: 64%, 4: 22%; and distribution of responses for *cognitive function:* 1: 1%, 2: 10%, 3: 63%, 4: 26%.

### 3.5. Statistical Evaluation of Questionnaire Domain Sub-Scores

#### 3.5.1. Descriptive Statistics

The sub-scores for the eight A to H domains of FUNCAP55 for ME/CFS respondents in Round 5 were generally fairly close to normal distribution over the range of possible scores (0 to 6), except for domain A (personal hygiene/basic functions), which was skewed towards high scores (see Supplementary Material File S2, Figure S1). The mean A to H sub-scores for FUNCAP55 and FUNCAP27 for ME/CFS respondents were significantly lower than those for HC respondents (Table 5). Mean A to H sub-scores were generally similar between FUNCAP55 and FUNCAP27 (Table 5). There were no statistically significant differences between men and women for the mean A to H FUNCAP55 domain sub-scores or total scores in Round 5 or Round 4.

##### Floor and Ceiling Effects

Ranges and floor and ceiling effects of the A to H sub-scores and total scores are presented in Table 6. For FUNCAP55, all A to H domain sub-scores had negligible (<5%) floor effects (with five < 1%). All but one domain (A 6%) had negligible ceiling effects (with six < 1%). For FUNCAP27, all domains had negligible (<5%) floor effects (with four < 1%), except D, which had a moderate effect (14.6%). All but one domain (A 7%) had negligible ceiling effects (with six < 1%). In summary, for FUNCAP55, 15/16 (94%) of F/C effects were negligible, with one being minor. Likewise, for FUNCAP27, 14/16 (86%) of F/C effects were negligible, with two being minor.

##### Correlations among Sub-Scores

The Pearson correlation coefficients between Round 5 FUNCAP55 vs. FUNCAP27 A to H sub-scores, including total scores (mean of A to H sub-scores), for ME/CFS respondents were as follows: A: 0.96; B: 0.98; C: 0.98; D: 0.93; E: 0.97; F: 0.98; G: 0.96; H: 0.96; total score: 0.99. Corresponding Pearson correlations between Round 4 FUNCAP55 and FUNCAP27 sub-scores, including total scores, for ME/CFS respondents were as follows: A: 0.97; B: 0.97; C: 0.98; D: 0.92; E: 0.97; F: 0.98; G: 0.97; H: 0.97; total score: 0.99. Correlations among FUNCAP55 sub-scores were generally high and very similar for Round 5 and Round 4 (Table 7). The same was true for FUNCAP27, although there were lower correlation coefficients (see Supplementary Material 2, Table S2).

#### 3.5.2. Reliability

##### Consistency

To assess the internal consistency (reliability) of the Round 5 FUNCAP55 and FUNCAP27 questionnaires’ A to H domain sub-scores and total scores, Cronbach´s alpha was assessed. These results are not directly comparable as a higher number of items in a domain inherently increases Cronbach’s alpha. For FUNCAP55/FUNCAP27, these were as follows: A: 0.92/0.77; B: 0.93/0.78; C: 0.91/0.83; D: 0.93/0.84; E: 0.91/0.80; F: 0.93/0.85; G: 0.87/0.78; H: 0.89/0.84; total score: 0.96/0.95. This corresponds to *good* (0.8 to < 0.9) or *excellent* (≥ 0.9) for all FUNCAP55 scores and for all but three FUNCAP27 scores, which were *acceptable* (0.7–0.79).

##### Round 6: FUNCAP27 Test–Retest Reliability

Round 6. The “Test” survey was open on 13–17 August 2023. The “Retest” survey was open 30 July to 2 August 2023. ME/CFS respondents were asked to complete FUNCAP27 twice, two weeks apart. For the test and retest, they entered a unique ID each time they took the survey, allowing for the pairing of their responses to the survey at Test and Retest. Of the 354 respondents that answered all FUNCAP items, 301 (85%) were younger than 60 years of age and were included, with a mean age of 43 (SD 0.6). For 234 respondents, diagnoses were made by a hospital consultant, and for 67, by a general practitioner. Of these, 5 ME/CFS respondents had *very severe*, 43 *severe*, 167 *moderate*, 80 *mild*, and 1 *better than mild* ME/CFS. Of the 300 people who responded to the question regarding their gender, 274 (91%) were women and 26 (9%) men. The IcCCs with 95% (CI) for the eight A to H domains and total scores were all excellent: A: 0.96 (0.95 to 0.97); B: 0.92 (0.90 to 0.93); C: 0.95 (0.93 to 0.96); D: 0.94 (0.93 to 0.95); E: 0.91 (0.89 to 0.93); F: 0.97 (0.96 to 0.97); G: 0.94 (0.93 to 0.96); H: 0.98 (0.97 to 0.98); total score: 0.98 (0.97 to 0.98).

#### 3.5.3. Validation

##### Known Group Validation—FUNCAP Sub-Scores vs. HCs

**Figure 1:**
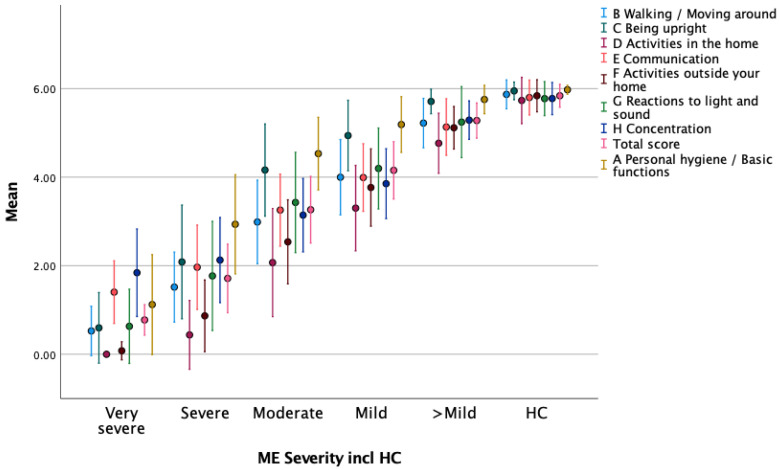
Round 5. Mean (+−1 SD) A to H sub-scores and total scores on the FUNCAP55 questionnaire for ME/CFS (*n* = 1263) respondents and healthy controls (HC, *n* = 178) according to ME/CFS severity degree which was as follows: *very severe* (*n* = 19), *severe* (*n* = 136), *moderate* (*n* = 733), *mild* (*n* = 360), *better than mild* (*n* = 15).

As described in Section 3.5.1 above, Round 5 ME/CFS respondents had significantly lower mean scores on all A to H domain sub-scores and significantly lower mean total scores for the FUNCAP55 and FUNCAP27 (Table 5) compared to HCs. This supports the “known group” validity of the FUNCAP. To further assess the “known group” validity of the FUNCAP, we determined how accurately it distinguished between the different ME/CFS ICC severity degrees based on patient reports from Round 5. The mean sub-scores for the eight A to H domains and total scores were compared between *very severe*, *severe*, *moderate*, *mild*, and *better than mild* ME/CFS subgroups. The mean A to H sub-scores and total scores were lowest for the most severely ill ME/CFS patient group, rising consecutively with less severe degrees of disease for all scales, with the highest mean sub-scores being in the HC group. This was the case for both the FUNCAP55 (Figure 1, Supplementary Material File S2, Table S3) and FUNCAP27 (see Supplementary Material File S2, Figure S2 and Table S3). For the FUNCAP55, we ran nine separate ANOVA analyses with Tukey post hoc tests, with the dependent variables being the A to H sub-scores and total scores and the independent variables being the five ME/CFS severity groups, excluding HCs. All ANOVA analyses were statistically significant (see Supplementary Material File S2, Table S3). The results of the post hoc analyses were all statistically significant, except for domains A: *mild* vs. *>mild* and H: *very severe* vs. *severe*. Corresponding ANOVA analyses were run for FUNCAP27, with the same results (see Supplementary Material File S2, Table S4) regarding statistically significant findings, except that there was, additionally, no statistically significant difference for sub-score D: *very severe* vs. *severe* in the post hoc analyses. Homogeneity-of-variance tests were statistically significant for all tests described here (all *p* values < 0.001). This supports our hypothesis of a monotonic relationship between sub-scores at different levels of severity of ME/CFS, such that mean scores at each successive level of severity were lower than the prior level, indicating a decline in FC with increasing level of MR/CFS severity. In summary, this supports both the “known group” validity of the FUNCAP and the facts that FUNCAP27 and FUNCAP55 FC sub-scores and total scores captured the range of ME/CFS severity.

##### Content Validation—FUNCAP Items vs. WHO ICF Functions

All FUNCAP55 items had content that was compatible with WHO ICF functions, except items A6, “Getting up and staying out of bed for approx. 1 h”, and F34, “Going on a necessary errand such as a doctor´s appointment” (Supplementary Material File S1, Table S1). The qualifier scale (response scale) in the WHO ICF is scored as follows: no impairment (none, absent, negligible), mild impairment (slight, low, …), moderate impairment (medium, fair,…), severe impairment (high, extreme, …), and complete impairment (total,…) (Supplementary Material File S1, Table S1).

## 4. Discussion

The present study describes a novel patient-informed questionnaire for assessing FC in ME/CFS patients, the FUNCAP, generated through repeated anonymous web-based survey rounds conducted in Norway. It comprises eight sub-domains, with mean scores for all individual items and domain sub-scores being significantly lower with increasing disease severity. Internal consistency was high and test–retest evaluation supported reliable questionnaire responses. The majority of FUNCAP domains also had negligible F/C effects. A separate, international English-language FUNCAP survey further supports its reliability and validity by exhibiting comparable findings regarding construct validity based on similar item domain and total mean scores as well as correlations between domain sub-scores compared to the Norwegian survey.

The evaluation of FUNCAP’s reliability, validity, and responsiveness is informed by COSMIN principles and definitions (COnsensus-based Standards for the selection of health Measurement Instruments) [12,28,32]. The excellent reliability of FUNCAP domain sub-scores and total scores is supported by both high Cronbach’s alpha levels, demonstrating good internal consistencies, and low measurement errors, demonstrated by excellent test–retest reliability. FUNCAP validity assessments were also positive. Our hypothesis of “known group” validity is supported. Good face validity is supported by extensive respondent feedback during item development, with 90% of respondents in the test–retest rounds stating the FUNCAP had a *good* or *very good* ability to assess their FC. Good structural validity and trans-cultural validity are also strengthened by very similar FUNCAP domain and total scores and domain correlations in both the Norwegian and the international (English-language) samples.

The content validity of FUNCAP items to assess FC is supported by all items, except two, having content that is in line with WHO ICF functions [24]. In general, FUNCAP items are, however, much more specific in their wording than WHO ICF functions. Therefore, based on consistent feedback from respondents in the FUNCAP development survey rounds, we think that the present WHO ICF functions, as they are described, are inadequate for assessing FC in ME/CFS patients. Additionally, the WHO ICF qualifier (response scale) is very different from that developed for the FUNCAP. Specifically, we maintain that the qualifier does not adequately take PEM into account as it does not assess the consequence of performing an individual activity. A recent study using a core set of items from WHO ICF to assess symptoms and FC in ME/CFS patients suggested that it needed a completely new category to assess PEM [33]. It also suggested several other additions and changes, many of them regarding ME/CFS symptoms, not FC [33].

Currently, there are no validated tools to accurately assess FC in ME/CFS patients. Cardiopulmonary testing, physical activity tracking, the SF-36 Physical Functioning subscale, and the Index of Independence in Activities of Daily Living have previously been used to validate the ICC classification of ME/CFS severity [34]. However, none of these accurately assess FC in ME/CFS patients, with the four-level ICC severity classification being an ambiguous FC scale. Not incorporating the consequences of PEM and the general lack of items central to FC in ME/CFS, such as cognitive and social activities, being upright, and being exposed to sound and light, are major shortcomings of the SF-36 [13]. The FUNCAP incorporates these activity categories. The widespread use of the SF-36 in many other diseases and in previous ME/CFS studies does not, in our opinion, overcome these disadvantages. It is important to note that the SF-36 is a questionnaire targeting general patient health status, not FC specifically. The broader generic Index of Independence in Activities of Daily Living targets very basic activities of daily living (ADLs), similar to domain A in the FUNCAP, supporting our item choices for that domain. However, this tool again does not consider PEM in the response scales [35]. A broader generic questionnaire, The Lawton Instrumental Activities of Daily Living Scale, has several items similar to FUNCAP’s activities in and outside the home (domains D and F), but, again, has the same problem with the response scale described for ADLs [36].

Among other questionnaires, the CFS-APQ [14] has previously been considered to be the best available tool for FC assessment in CFS and ME/CFS patients [12]. Its content and construct validity were judged to be moderate, based on study populations using the Fukuda CFS inclusion criteria [6]. This is problematic since these criteria do not require PEM to be present. Furthermore, the consequences of PEM are not adequately incorporated into the items of the questionnaire. It shares the problem seen in other questionnaires in that when inquiring whether patients can perform a specific activity or not, it fails to consider the potential consequences on PEM. This may lead to some indicating that they can perform the activity, disregarding the induced PEM, while others may take PEM into account and report an inability to perform the activity. We maintain that the CFS-APQ identifies many important restrictive activities for ME/CFS patients, but to a lesser extent than the FUNCAP. Importantly, the CFS-APQ does not incorporate the consequences of PEM on FC, which are central to the approach used in the FUNCAP. Finally, a questionnaire that does include items relating to PEM is the DePaul Post-Exertional Malaise Questionnaire (DPEMQ), developed with feedback from ME/CFS patients [37]. Of special interest for the present study, the authors state that “There needs to be items on questionnaires that assess items such as what would happen if a patient were to engage in exertion producing activities, as well as if they are pacing to reduce symptom exacerbation” [37]. This echoes feedback from respondents in Round 1 of the present study and its implementation into the final FUNCAP response scale.

An issue with other outcome assessment tools used for ME/CFS is the presence of F/C effects [38]. By developing a patient-informed questionnaire using extensive feedback from ME/CFS patients, we have been able to create a questionnaire with low to negligible F/C effects. The significance of this is that the FUNCAP can accurately capture functional capacity in both mild and very severe ME/CFS patients. This is further supported by domain scores and total scores being able to significantly differentiate between the ICC severity categories.

An advantage of developing the FUNCAP in Norway was the well-developed, universal healthcare system with national guidelines for the diagnosis and management of ME/CFS. This makes an accurate diagnosis of ME/CFS more likely, although it is still probably underdiagnosed in Norway. Using anonymous, web-based surveys facilitated broad participation since this required minimal respondent activity and maximum flexibility in completing the questionnaire. This is particularly important for the most severely affected patients. We also believe respondents were motivated to participate in consecutive survey rounds as they could directly observe how their feedback resulted in the changes made to the questionnaire. The existence of a large and well-organized ME/CFS patient organization and an extensive pre-existing social media group facilitated study recruitment.

Another strength of the present study is the consistency in responses, with very similar FUNCAP55 item mean values and standard deviations in two large, separate samples with similar distributions of ME/CFS severity—the Norwegian and the international English. This supports the reliability and generalizability of the FUNCAP.

With the increasing recognition of the large number of people affected by long COVID-19 [39], many of whom have PEM [9,40], there is a growing need for having effective assessment tools targeting FC while taking PEM into account. We hope that the FUNCAP can contribute to this.

A limitation of the present study is that we did not follow the recommended method for translating a PROM using two independent translators and back-translators, as we used only one [41]. This may have resulted in some small differences in items from the Norwegian to the English version. However, we believe any such differences are minor given the mainly factual item content. Another limitation is that we relied on self-reporting of the diagnosis of ME/CFS, as patient medical records were not available. Using this strategy is also very likely to have been a major cause of achieving such high sample sizes. Still, this may reduce the generalizability of our findings compared to an ideal study. We do not interpret the very high female-to-male ratio among respondents in the present study as skewed participation regarding gender, as similar findings have been described previously [42]. It is also worth noting that the total scores in the present study may be imprecise as a reliable indicator of “total FC” in ME/CFS patients. Some FUNCAP domains may be more important than others in this regard. Also, the concept of “total FC” is dependent on personal and societal settings of individuals and what aspects of FC are most important to them. For example, patients with extensive orthostatic intolerance but relatively high FC regarding concentration/cognition could conceivably be employed to some degree if working lying down is an option.

We excluded those aged above 60 years in the data analysis (not in the questionnaire item development) based on indications that the ME/CFS phenotype may differ with higher age [29]. An additional reason was that the frequency of co-morbidities reducing FC would likely increase with increasing age potentially reducing the interpretability of our analyses. We see this as both a strength and a limitation. It points to the possibility of future analyses regarding FC specifically in the older age group, which could include analyses incorporating the relationship of age with FC in ME/CFS respondents including co-morbidities, disease severity, onset, and duration. As we did not collect data regarding co-morbidities in ME/CFS respondents, if present, regardless of age, these may have reduced a respondent’s FC and influenced their FUNCAP item responses.

It is important to note that FUNCAP does not attempt to assess quality of life or the range of ME/CFS symptoms, which include fatigue, pain, nausea, and unrefreshing sleep. The assessment of such aspects would be important an addition to FC assessment, not only in clinical work, but also in research, for example, targeting the effectiveness of interventions or treatments.

Finally, an unavoidable limitation is responses from caregivers when the target respondent was too ill to answer, which may have resulted in inaccurate responses. A previous study of individuals aged 65 years and older recovering from hip fracture found support for observable activities, as most FUNCAP items are, being similarly rated by proxies and subjects [43]. Related to this is the difficult question of who can provide the most accurate response regarding ME/CFS FC. This may not necessarily be the person with ME/CFS depending on the domain/item. Observations made by a person close to the patient may provide a more accurate and precise answer in some instances. Having as positive a view of one’s total life situation with ME/CFS as possible may be a useful coping strategy but can lead to ME/CFS respondents scoring FUNCAP items too optimistically.

## 5. Conclusions

Our findings support the utility of the FUNCAP55 as an effective, reliable, and valid tool for assessing FC in ME/CFS patients. It may prove valuable in clinical diagnostic work or follow-up consultations and for assessing liability for disability benefits. Importantly, it may reveal the actual FC of people with ME/CFS, helping in the provision of improved day-to-day care for the more severely affected ME/CFS patients and in the adjusting of expectations and demands from social surroundings to a more reasonable level for the less severely affected. The aspect of delayed PEM after overstepping one´s maximum regular FC level highlights this. The shorter version, FUNCAP27, may prove valuable in research on the efficacy of treatments and interventions in ME/CFS patients. The strikingly similar findings gathered in the present study on all aspects of FUNCAP responses in two separate surveys in different countries support the conclusion of recent extensive reviews, namely that ME/CFS is a distinct disease entity [3]. We hope that the FUNCAP can contribute to a better general societal recognition of the severe and often hidden debilitating nature of ME/CFS and that it may be of use in assessing FC in other diseases, such as long COVID-19.

## Figures and Tables

**Table 1:** Number of respondents who answered all FUNCAP items (all ages) with severity degrees, gender, and age below 60 years across all study rounds. Numbers of respondents (per cent). Not all participants answered questions about ME/CFS severity.

	Round 1	Round 2	Round 3	Round 4	Round 5	Round 6
Total respondents	290	435	536	1945 ^1^	1463 ^1^	353 ^1^
Very severe	9/256 (4)	7/431 (2)		50 (2.6)	22 (1.5)	1 (0.3)
Severe	40/256 (16)	58/431 (13)		395 (20)	155 (11)	11 (26)
Moderate	160/256 (63)	274//431 (64)		1062 (55)	847 (58)	200 (57)
Mild	47/256 (19)	92/431 (21)		367 (19)	425 (29)	51 (14)
Better than mild				66 (3.4)	16 (1.3)	11 (3)
Female	231/253 (92)	385/428 (90)	476/536 (89)	1614/1915 (84)	1269/1450 (88)	318/353 (90)
Male	21/253 (8)	43/428 (10)	60/536 (11)	301/1915 (16)	181/1450 (12)	35/353 (10)
Age under 60 years	237/253 (94)	401/429 (93)	511/536 (95)	1387/1940 (71)	1263/1463 (86)	301/353 (10)

^1^ Respondents that had complete FUNCAP55 item responses. Among these, 1940 indicated severity degree and age.

**Table 2:** Round 5 (Norwegian) ME/CFS and healthy control (HC) respondents’ and Round 4 (international/English) ME/CFS respondents’ characteristics for those completing the questionnaires. Only respondents < 60 years of age are included. Numbers of respondents (per cent).

	ME/CFS Round 5 (*n =* 1263)	HC Round 5 (*n =* 178)	ME/CFS Round 4 (*n* = 1387)
Female/Male ^1^	1102/149 (88)	118/56 (68)	1157/209 (85/15)
Very severe ME/CFS	19 (2)		44 (3.2)
Severe ME/CFS	136 (11)		299 (22)
Moderate ME/CFS	733 (58)		752 (54)
Mild ME/CFS	360 (29)		255 (18)
Better than mild ME/CFS	15 (1)		37 (2.7)
Age < 19 years	48 (4)	3 (2)	15 (1.1)
Age 20–39 years	420 (33)	67 (38)	440 (32)
Age 40+ years	795 (63)	108 (61)	932 (67)
Onset < 15 years	194 (15)		169 (12)
Onset 16–29 years	424 (29)		533 (38)
Onset 30+ years	646 (45)		684 (49)
Duration < 5 years	143 (12)		289 (21)
Duration 6–15 years	666 (56)		582 (42)
Duration 16+ years	372 (31)		516 (37)
Disability benefits	1038 (82)	5 (3)	
Parttime < 50% work/education	120 (10)	4 (2)	
Parttime 50–100% work/education	33 (3)	11 (6)	
Full time work/education	30 (2)	149 (84)	
Stay at home	-	6 (3)	
None of the above	42 (3)		

^1^ Twelve ME/CFS and four HC respondents in Round 5 and twenty-one respondents in Round 4 preferred not to state their gender.

**Table 3:** Rounds 5 and 4. Mean (SD) item scores (0 to 6) and mean differences in scores for the 55 items in the main Norwegian Round 5 FUNCAP55 for ME/CFS (ME, *n* = 1236), healthy controls (HCs, *n* = 178) and the international Round 4 (English language, *n* = 1387) respondents. The scoring scale was as follows: 0: I cannot do this. 1: My capacity will be severely reduced for at least three days. 3: I can do little else on the same day. 4: I must limit other activities on the same day. 5: This rarely affects other activities. 6: Unproblematic—does not affect other activities. The numbers in parentheses after each item number indicate the number the same item had in FUNCAP27. Round 4 item wordings differ somewhat from those in Round 5, which are in Table 3 (see Supplementary Material File S1).

	Round 5					Round 4	
	ME		HC		HC-ME	ME	
Items	Mean	SD	Mean	SD	Mean Diff.	Mean	SD
A1 (1) Using toilet (not bedpan or bedside commode)	5.3	0.9	6.0	0.1	0.7	5.1	1.1
A2 Brushing your teeth without assistance	5.2	1.0	6.0	0.0	0.8	5.2	1.0
A3 Showering seated, with assistance	4.6	1.6	6.0	0.5	1.4	4.4	1.6
A4 Showering seated, without assistance	4.2	1.6	6.0	0.1	1.8	4.1	1.7
A5 (2) Showering standing up	3.4	1.8	6.0	0.2	2.6	3.3	2.0
A6 Getting up and staying out of bed for approx. 1 h	4.5	1.4	6.0	0.1	1.5	4.2	1.6
A7 (3) Getting dressed in regular clothes	4.8	1.2	6.0	0.1	1.2	4.7	1.2
B8 (4) Walking a short distance indoors, from one room to another	4.9	1.1	6.0	0.1	1.1	4.6	1.3
B9 Walking a short continuous distance, approx. 100 m (length of a football field), in- or outdoors	4.0	1.6	6.0	1.0	2.0	3.7	1.8
B10 (5) Walking between approx. 100 m and 1 km on level ground (length of 1 to 10 football fields)	3.0	1.7	6.0	0.2	3.0	2.6	1.9
B11 Going for a longer walk. Approx. 1 km (0.6 mile), mostly level ground	2.4	1.7	5.9	0.3	3.5	1.9	1.8
B12 Going for a longer walk. Approx. 1 km (0.6 mile), hilly or varied terrain	1.8	1.6	5.8	0.5	4.0	1.3	1.6
B13 (6) Physical activity with increased heart rate, for approx. 15 min	1.4	1.4	5.7	0.8	4.3	1.5	1.6
B14 Physical activity with increased heart rate, for approx. ½ h	0.7	1.2	5.4	1.1	4.7	0.9	1.4
C15 (7) Sitting in bed for approx. 30 min	5.2	1.2	6.0	0.1	0.8	5.0	1.4
C16 Physical activity with increased heart rate, for approx. ½ h	4.8	1.4	6.0	0.4	1.2	4.5	1.6
C17 (8) Sitting in an upright chair (dining chair) with feet on floor for approx. 2 h	3.2	1.9	5.9	0.5	2.7	2.8	2.1
C18 (9) Standing up for approx. 5 min, e.g., while queuing or while cooking	3.9	1.6	6.0	0.1	2.0	3.9	1.8
C19 Standing up for a long time—approx. ½ h	2.5	1.8	5.9	0.4	3.4	2.2	1.9
D20 Light housework (dusting, tidying etc.) for approx. ½ h continuously	2.9	1.6	5.9	0.4	3.0	2.7	1.8
D21 (10) Heavier housework (washing floors, vacuuming etc.) for approx. ½ h continuously	1.9	1.5	5.7	0.6	3.8	1.8	1.7
D22 Laundry (sorting, hanging up to dry and folding)	3.3	1.5	5.9	0.3	2.6	3.0	1.7
D23 Making a simple cold meal, such as a sandwich or cereal	4.8	1.3	6.0	0.1	1.2	3.3	1.8
D24 Cooking a simple hot meal	4.1	1.4	5.9	0.2	1.8	4.3	1.5
D25 (11) Cooking a complicated meal from scratch, approx. 1 h of preparation	2.6	1.6	5.8	0.5	3.2	1.9	1.7
E26 Speaking a few words	5.6	0.8	6.0	0.1	0.4	5.5	0.9
E27 (12) Having a conversation for approx. 5 min	5.2	1.0	6.0	0.1	0.8	5.0	1.1
E28 Having a conversation for approx. ½ h	4.0	1.3	5.9	0.3	1.9	3.9	1.4
E29 Writing a short message by hand	5.3	1.0	6.0	0.1	0.7	5.2	1.0
E30 (13) Participating in a conversation with three people for approx. ½ h	3.1	1.5	5.9	0.4	2.7	3.1	1.6
E31 Socializing with friends for approx. 1 h	2.7	1.4	5.8	0.5	3.1	2.3	1.6
E32 (14) Participating in a dinner party, party or family event	1.7	1.2	5.6	0.8	3.9	1.8	1.4
F33 (15) Stepping right outside your home	4.5	1.5	6.0	0.1	1.5	4.2	1.8
F34 Going on a necessary errand, such as a doctor’s appointment	3.0	1.4	5.9	0.4	2.9	2.9	1.5
F35 (16) Going to a shop for groceries	2.9	1.5	5.9	0.4	3.0	2.2	1.7
F36 Doing enjoyable leisure activities, such as going to a café, non-essential shopping etc.	2.5	1.5	5.9	0.3	3.4	2.4	1.6
F37 Riding as a passenger in a car for approx. 15 min	4.3	1.5	6.0	0.2	1.7	3.9	1.7
F38 (17) Using public transport (bus or train)	2.4	1.8	5.9	0.4	3.5	2.1	1.9
F39 (18) Participating in organized leisure activities such as classes, sports etc.	1.0	1.4	5.6	0.8	4.6	1.1	1.5
G40 Staying in a room with dim lighting for approx. 1/2 h	5.4	0.9	6.0	0.1	0.6	5.5	1.0
G41 (19) Staying in a room with normal lighting, without sunglasses, for approx. 1 h	5.0	1.4	6.0	0.1	1.0	4.9	1.5
G42 (20) Staying outdoors in daylight without sunglasses for approx. 2 h	3.2	1.9	5.8	0.5	2.6	3.4	2.1
G43 Staying in an environment with the sound of a few people in quiet conversation	3.9	1.4	6.0	0.2	2.1	4.2	1.6
G44 (21) Staying in a noisy environment, (shopping mall, café or open plan office) for approx. 1 h	2.2	1.4	5.5	0.7	3.4	2.6	1.8
G45 Going to a cinema, concert etc. with high noise levels	1.6	1.4	5.6	0.8	4.0	1.7	1.7
H46 (22) Reading a short text, such as a mobile phone text message	5.4	0.9	6.0	0.1	0.6	5.4	0.9
H47 Reading fiction/light reading	3.3	2.0	5.9	0.3	2.6	3.5	2.0
H48 (23) Reading and understanding a non-fiction text, such as an official document one A4 page long	3.1	1.7	5.8	0.5	2.7	4.0	1.6
H49 Performing simple mental arithmetic	4.1	1.7	5.9	0.5	1.8	4.2	1.7
H50 Writing short messages on a smartphone or tablet	5.2	0.9	6.0	0.2	0.8	5.0	1.0
H51 (24) Using social media to stay in touch with others	4.6	1.2	5.9	0.3	1.4	4.6	1.3
H52 Watching TV (series, news)	4.5	1.2	6.0	0.2	1.5	4.5	1.4
H53 (25) Focusing on a task for approx. 10 min continuously	3.8	1.5	5.9	0.3	2.1	4.3	1.3
H54 (26) Focusing on a task for approx. 2 h continuously	1.9	1.6	5.6	0.8	3.7	2.4	1.8
H55 (27) Managing a full working day (non-physical work such as office work, classes, or lectures)	0.6	1.1	5.5	0.9	4.8	0.8	1.4

**Table 4:** Round 5 FUNCAP55 respondent evaluation, *n* (%), (*n* = 1263).

	Strongly Agree	Agree	Disagree	Strongly Disagree
Easy to understand	501 (40)	740 (59)	20 (2)	2 (0.2)
Easy to know what to answer to the questions	283 (22)	808 (64)	164 (13)	8 (0.6)
Gave a correct picture of my illness-situation	292 (23)	877 (69)	91 (7)	3 (0.2)
Needed help from others to answer	66 (5)	79 (6)	394 (31)	724 (57)
Needed several breaks when filling out	69 (6)	323 (26)	528 (42)	343 (27)

**Table 5:** Round 5. FUNCAP55 and FUNCAP27: Mean sub-scores (SD) for the eight A to H domains and total scores (mean of A to H sub-scores) for the ME/CFS and HC respondents. The scoring scale was as follows: 0: I cannot do this. 1: My capacity will be severely reduced for at least three days. 3: I can do little else on the same day. 4: I must limit other activities on the same day. 5: This rarely affects other activities. 6: Unproblematic—does not affect other activities. All mean differences were statistically significant, with *t*-test *p*-values < 0.009 with Bonferroni correction.

**FUNCAP55 A to H domains**	**ME/CFS** **(*n* = 1263)**	**HC** **(*n* = 178)**	**Mean Difference** **95% CI**
A Personal hygiene/basic functions	4.6 (1.1)	6.0 (0.1)	−1.4 (−1.6 to −1.2)
B Walking/moving around	2.3 (1.1)	5.1 (0.3)	−2.8 (−3.0 to −3.6)
C Being upright	3.9 (1.4)	5.9 (0.2)	−2.0 (−2.2 to −1.8)
D Activities in the home	3.3 (1.3)	5.9 (0.3)	−2.6 (−2.8 to −2.4)
E Communication	3.9 (1.0)	5.9 (0.3)	−1.9 (−2.1 to −1.8)
F Activities outside your home	3.0 (1.3)	5.9 (0.3)	−2.9 (−3.1 to −2.7)
G Reactions to light and sound	3.5 (1.1)	5.8 (0.3)	−2.3 (−2.4 to −2.1)
H Concentration	3.7 (1.0)	5.8 (0.3)	−2.2 (−2.3 to −2.0)
Total score (mean of A–H sub-scores)	3.5 (1.0)	5.8 (0.2)	−2.3 (−2.4 to −2.1)
**FUNCAP27 A to H domains**	**ME/CFS** **(*n* = 1263)**	**HC** **(*n* = 178)**	**Mean Difference** **95% CI**
	4.5 (1.1)	6.0 (0.1)	−1.5 (−1.6 to −1.3)
B Walking/moving around	3.1 (1.2)	5.9 (0.3)	−2.8 (−2.9 to −2.6)
C Being upright	4.1 (1.4)	5.9 (0.2)	−1.8 (−2.0 to −1.6)
D Activities in the home	2.2 (1.4)	5.7 (0.5)	−3.5 (−3.7 to −3.3)
E Communication	3.3 (1.0)	5.8 (0.4)	−2.5 (−2.6 to −2.3)
F Activities outside your home	2.7 (1.3)	5.8 (0.4)	−3.1 (−3.3 to −2.9)
G Reactions to light and sound	3.4 (1.3)	5.8 (0.4)	−2.3 (−2.5 to −2.1)
H Concentration	3.2 (1.0)	5.8 (0.4)	−2.5 (−2.7 to −2.4)
Total score	3.3 (1.1)	5.8 (0.3)	−2.5 (−2.7 to −2.3)

**Table 6:** Round 5 ME/CFS respondents (*n* = 1263). FUNCAP55 and FUNCAP27: Ranges and floor and ceiling effects for the eight A to H domains and total scores (mean of A to H sub-scores) for ME/CFS and HC respondents. The scoring scale was as follows: 0: I cannot do this. 1: My capacity will be severely reduced for at least three days. 3: I can do little else on the same day. 4: I must limit other activities on the same day. 5: This rarely affects other activities. 6: Unproblematic—does not affect other activities.

	Range	FUNCAP55 Floor	Ceiling	Range	FUNCAP27 Floor	Ceiling
A Personal hygiene/Basic functions	0–6	0.2	6.0	0–6	0.6	7.1
B Walking/Moving around	0–5.3	1.0	0	0–6	1.0	0.4
C Being upright	0–6	1.4	1.5	0–6	1.5	4.2
D Activities in the home	0–6	2.9	0.2	0–6	14.6	0.2
E Communication	0–6	0.2	0.3	0–6	0.6	0.3
F Activities outside your home	0–6	2.3	0.1	0–6	3.6	0.1
G Reactions to light and sound	0–6	0.6	0.5	0–6	2.2	0.6
H Concentration	0–6	0.2	0.2	0–6	0.2	0.2
Total Score	0–5.9	0.1	0	0–6	0.1	0.1

**Table 7:** Correlations among the eight A to H FUNCAP55 domain sub-scores (in addition to total scores, i.e., the mean of the eight A to H sub-scores) for Round 5 and 4 ME/CFS respondents.

**Round 5. Norwegian (*n* = 1263):**									
Domains, ME/CFS respondents	A	B	C	D	E	F	G	H	TS
A. Personal hygiene/basic functions	1	0.76	0.82	0.82	0.74	0.78	0.72	0.64	0.88
B. Walking/moving around		1	0.77	0.82	0.72	0.83	0.70	0.65	0.88
C. Being upright			1	0.84	0.79	0.80	0.75	0.68	0.91
D. Activities in home				1	0.79	0.86	0.76	0.71	0.93
E. Communication					1	0.81	0.81	0.80	0.90
F. Activities outside your home						1	0.81	0.73	0.93
G. Reactions to light and sound							1	0.74	0.88
H. Concentration								1	0.82
TS. Total score (mean of A-H sub-scores)									1
**Round 4. International/English (*n* = 1387):**									
Domains, ME/CFS respondents	A	B	C	D	E	F	G	H	TS
A. Personal hygiene/basic functions	1	0.73	0.82	0.80	0.72	0.77	0.71	0.65	0.88
B. Walking/moving around		1	0.76	0.81	0.68	0.83	0.66	0.58	0.86
C. Being upright			1	0.72	0.76	0.80	0.73	0.66	0.91
D. Activities in home				1	0.76	0.86	0.73	0.69	0.92
E. Communication					1	0.82	0.79	0.82	0.88
F. Activities outside your home						1	0.78	0.70	0.93
G. Reactions to light and sound							1	0.73	0.86
H. Concentration								1	0.81
TS. Total score (mean of A-H sub-scores)									1

## Data Availability

Selected data presented in this study are available on reasonable request from the corresponding author. The authors have a website, www.funcap.no, under development that will contain relevant issues related to the clinical and research use of the FUNCAP questionnaire. It will provide continuously updated information.

## Supplementary Materials

The following supporting information can be downloaded at: https://www.mdpi.com/article/10.3390/jcm13123486/s1. Supplementary Material File S1: Table S1. FUNCAP55 items and the nearest, corresponding WHO International classification of functioning, disability and health—ICF, function [4]. More detailed description of each function is provided in the WHO ICF manual. Supplementary Material File S2: Table S2. Correlations among FUNCAP27 A to H sub-scores (and Total Score, i.e., the mean of the eight A to H sub-scores) for the ME/CFS Round 5 and 4 respondents. All Pearson Correlation *p*-values were < 0.008 (with Bonferroni correction). Figure S1. Histograms of the eight A to H domain sub-scores for Round 5 FUNCAP55 ME/CFS respondents (*n* = 1263). Figure S2. Round 5. Mean (± 1 SD) A to H sub-scores and Total Score on the FUNCAP27 questionnaire for the ME/CFS (*n* = 1263) and healthy controls (HC, *n* = 178) respondents according to ME/CFS severity degree which was as follows: *Very severe* (*n* = 19), *severe* (*n* = 136), *moderate* (*n* = 733), *mild* (*n* = 360), *better than mild* (*n* = 15). Table S3. Round 5 mean (SD) FUNCAP55 A to H domain sub-scores according to ME/CFS severity degrees. HC were not included in statistical analyses. ANOVA analyses for each domain were all statistically significant with *p* < 0.001. Post hoc tests (Tukey) between groups were all statistically significant with *p* < 0.001 (B: *Very severe* vs. *severe p* = 0.03. C: *Mild* vs. *>mild p* = 0.009. E: *Very severe* vs. *severe p* = 0.003) except: A: *Mild* vs. *>mild p* = 0.1 and H: *Very severe* vs *severe p* = 0.08. The numbers in each severity were: *Very severe* (19), *severe* (*n* = 136), moderate (*n* = 733), *mild* (*n* = 360) and *better than mild* (*n* = 15). Table S4. Round 5 mean (SD) FUNCAP27 A to H domain sub-scores according to ME/CFS severity degrees. HC were not included in statistical analyses. ANOVA analyses for each domain were all statistically significant with *p* < 0.001. Post hoc tests (Tukey) between groups were all statistically significant with *p* < 0.001 (C: *Mild* vs. *>mild p* = 0.03. E: *Very severe* vs. *severe p* = 0.04. F: *Very severe* vs. *severe p* = 0.003. G: *Mild* vs. *>mild p* = 0.002) except: A: *Mild* vs. *>mild p* = 0.06. D: *Very severe* vs. *severe p* = 0.48. H: *Very severe* vs. *severe p* = 0.63. The numbers in each severity were: Very severe (19), severe (*n* = 136), moderate (*n* = 733), mild (*n* = 360) and better than mild (*n* = 15). FUNCAP Questionnaires: In Supplementary Material File S2: The complete, respondent-ready Norwegian and English FUNCAP55 and FUNCAP27 questionnaires.
