# Supplementary material for: Assessing Functional Capacity in Myalgic Encephalopathy/Chronic Fatigue Syndrome: A Patient-Informed Questionnaire
Source: J Clin Med. 2024 Jun 14;13(12):3486. doi: 10.3390/jcm13123486 (PMC11204454; doi:10.3390/jcm13123486)
Supplement: Supplementary file 1 [file jcm-13-03486-s001.zip › jcm-2965438 - Supplementary material S1 .pdf]

Supplemental Material S1. FUNCAP

Translation from Norwegian to English of Rounds 1-3 was done by study authors (KS, TS):

Item and FUNCAP domain/ subscore sources:

N = Norsk Funksjonsskjema (Norwegian Function Questionaire) [1]
C = CFS-APQ [2]
A = ADLS [3]
T = TSSS [3]
X = Novel, written by study authors (KS, TS)

Time and distance descriptions in items were partly taken from the above N, C, A sources. Some time and distance descriptions were added by the authors, and some were modified based on survey responses.

Specific sources: Walking 1 km: N. Standing 1 hour: C. Sitting 2 hours: C. Computer work 30 minutes: C. Read 30 minutes: C. Conversation of 5 minutes: A.

Round 1

An introduction gave the reason for asking for help from ME/CFS patients in developing the questionnaire.

We asked about:
Gender (male, female, prefer not to say)
Age-group (< 20, 20 – 30, 31 - 40, 41 – 50, 51 – 60, 61 – 70, > 70)
ME/CFS severity degree (ICC criteria)

They were informed:
"It is best to answer the questionnaire together with a person who is with you regularly".
They were asked to answer according to how they had been the last seven days.

Response scale:
This scale was from ADLS [3]
How often during the last week have you done the following activities?
Response scale: Never/ 1 day/ 2-3 days/ 4-6 days/ Every day.
The Response scale was written by study authors (KS, TS)

1. Personal hygiene
1. Used WC without assistance (A)
2. Brushed teeth without assistance (X)
3. Taken a shower (A)
4. Gotten dressed in regular clothes and got out of bed (A, N)

2. Walk, stand, being upright
5. Stood for 5 minutes or more (for example queuing/ cooking) (N)
6. Sat in an upright chair with feet on the floor for more than 10 minutes (N)
7. Walked a short distance, up to approximately 100 m (N)
8. Walked a short distance on level ground approximately 100 m to 1 km (N)
9. Taken a longer walk (more than 1 km) (N)
10. Walked stairs (at least one floor) (N)

11. Light activities, e.g. tidying, dusting (C)
12. Heavier activities, f ex washing floors, vacuuming (N)
13. Doing laundry (C)
14. Made a simple sandwich (N)

4. Communication
15. Been able to talk at all (A)
16. Had a conversation for more than 5 minutes (A)
17. Had a conversation for more than 30 minutes (A)
18. Written short messages or texted (N)
19. Been able to participate in a conversation with several people (N)

5. Activities out of home
20. Necessary activities such as a doctor´s appointment (X)
21. Pleasant activities outside home, e.g. visiting a café, visiting friends (N)
22. Gone to shop to get groceries (N)
23. Been a passenger in a car (N)
24. Used public transport (bus or train) (N)
25. Participated in organized leisure activities (N)

Answer: How often during the last week have you experienced the following:
Response scale for 6, 7, 8, 9: Never/ Rarely/ Sometimes/ Often/ Very often

6. Sensory input
26. Problems tolerating daylight outside (had to use sunglasses because of discomfort) (T)
27. Problems tolerating regular light indoors (had to use sunglasses because of discomfort) (T)
28. Experienced normal sound level to be uncomfortable (T)
29. Used earplugs / earmuffs to protect myself against sound (X)

7: PEM – exertion induced symptom increase
30. Become clearly worse and had to do less the day(s) after having done definitely more than you usually do (X)
31. Suddenly felt a total lack of energy (X)
32. Had to lie down and rest once during the day (X)
33. Had to lie down and rest several times during the day (X)
34. Not been able to complete a task you started because you became too physically exhausted (X)
35. Not been able to complete a task you started because your ability to concentrate disappeared (X)
36. Had to choose between two tasks, because both would be too much (f ex shower and shop the same day) (X)
37. Experienced a loss of concentration after physical activity (X)
38. Experienced having less physical capacity after mental activity (X)

8. Concentration and attention
39. Experienced reduced memory (N)
40. Experienced reduced attention and concentration (N)
41. Had problems finding words or said a wrong word (X)
42. Experienced problems with simple mental arithmetic (X)
43. Had problems remembering oral instructions (N)

44. Had good enough concentration to read a book (N)
45. Had concentration to watch a TV-series (N)

Feedback

Respondents were also asked to tell how well they felt they were able to communicate their FC when it came to physical function, concentration, memory, sensitivity to light and sound, PEM, orthostatic intolerance, and whether asking about activity over the last week was acceptable.

Respondents were also asked to suggest improvements to the form in free text.

Round 2

An introduction gave the reason for asking for help from ME/CFS patients in developing the questionnaire.

We asked about:
Gender (male, female, prefer not to say)
Age-group (< 20, 20 – 30, 31 - 40, 41 – 50, 51 – 60, 61 – 70, > 70)
ME/CFS severity degree (ICC criteria)

They were informed:
"It is best to answer the questionnaire together with a person who is with you regularly".

Response scale

What would it cost you in terms of effort and possible symptom exacerbation to perform these activities? How would it affect your ability to perform other activities?

Scale (all items)

1. I cannot do this activity I cannot do this activity without severe deterioration
2. Very much- can do nothing else the same day or days after
3. A lot- can do nothing else the same day
4. Fairly much – reduce activity level the same day/ have to take extra rest before or afterwards
5. Some – but rarely affects other activities
6. Unproblematic – does not affect other activities

A. Personal hygiene / basic functions
1. Use WC without assistance (A)
2. Brush teeth without assistance (X)
3. Take a shower without assistance (A)
4. Get out of bed and be around the house for more than one hour (X)
5. Put on regular clothes and get out of bed (A, N)

B. Walking and other physical activity
6. Walk a short distance indoors between bedroom / bathroom or kitchen (N)
7. Walk a continuous distance, approximately 100m (indoors or outdoors) (N)
8. Walk on level ground approximately 100 m to 1 km (N)
9. Go for longer walk (more than 1 km) mostly flat (N)
10. Go for a longer walk, (more than 1 km) in hilly/ varied terrain (N)

11. Physical activity giving increased heartrate for at least 15 min (N)
12. Physical activity with mostly continuous high pulse for ½ hour (N)

C. Being upright
13. Sit in bed (A)
14. Sit in in an upright chair (dining chair) with feet on floor for at least 10 minutes (N)
15. Sit in an upright chair with feet on floor for several hours (N)
16. Stan for approximately 5 minutes (for example queuing/ cooking) (N)
17. Stand for a long time, 30 minutes or more (N)

D. Activities at home
18. Light activity, f ex tidying/ dusting, for at least 30 min continuously (N)
19. Heavier activities, f ex washing floors/ vacuuming (N)
20. Doing laundry (C)
21. Fixing a simple sandwich (N)
22. Preparing a simple hot meal/ dinner (N)
23. Preparing a complicated meal from scratch (more than an hour of preparation) (N)

E. Communication and being with other people
24. Saying a few words (A)
25. Having a conversation for at least 5 minutes (A)
26. Having a conversation for more than 30 minutes (A)
27. Writing short messages (N)
28. Participating in a conversation with several people (N)
29. Having a visitor or visiting a friend (C, A)
30. Participating in a dinner party/ birthday party/ big family event (C)

F. Activities outside the home
31. Being right outside the home, going to the mailbox or similar (A)
32. Doing necessary errands, like doctor's appointment (X)
33. Doing pleasant activities outside the home, eg. visiting a café, shopping, visiting friends (N)
34. Going to the shops to get groceries (N)
35. Being a passenger in a car (N)
36. Using public transport (busses/trains) (N)
37. Participating in organized leisure activities (N)

G. Reactions to light and sound
38. Being in a room with dimmed lighting (T)
39. Being in normal indoor lighting (without sunglasses) (T)
40. Being outdoors in daylight without sunglasses (T)
41. Tolerating normal sound levels at home (T)
42. Tolerating normal outside sound levels (T)
43. Tolerating noisy surroundings (shopping mall, café) (X)
44. Going to a cinema or concert with loud sound (X)

H. Concentration
45. Reading a short text, e. g. an SMS (N)
46. Reading ½ A4 page continuously (N)
47. Reading a longer text (book, long article (N)
48. Doing simple mental arithmetic (X)
49. Writing short messages on a smartphone or tablet (A)

50. Using social media to stay in touch with others (A)
51. Watching TV (series, news) (N)
52. Focusing on a task for 10 minutes continuously (N)
53. Focusing on a task for at least 2 hours continuously (N)
54. Focusing for a full working day (office work) (N)

After the end of the questionnaire items, the respondents were asked to rate how well the questionnaire illustrated various aspects of their reduced functional capacity on a 5-point Likert scale: Very bad, bad, neither good nor bad, good, very good.

We asked about: Physical capacity, Concentration, Memory, Hypersensitivity to sound and light, Post exertional malaise (exertion induced symptom increase), Orthostatic intolerance (dizzy or unwell while sitting or standing up), How much you do in the course of a day, That you had to prioritize one activity over another, Variation in capacity/ disease fluctuation.

Feedback

Respondents were also asked to suggest improvements to the form in free text.

Round 3

An introduction gave the reason for asking for help from ME/CFS patients in developing the questionnaire.

We asked about:
Gender (male, female, prefer not to say)
Age-group (< 20, 20 – 30, 31 - 40, 41 – 50, 51 – 60, 61 – 70, > 70)

They were informed:
"It is best to answer the questionnaire together with a person who is with you regularly".

Answer with an average day in mind. "It is best to answer the questionnaire together with a person who is with you regularly". "A close person can answer on behalf of someone with ME/CFS who is too sick to answer by themselves".

Respondents were also asked to provide demographic information as in round 2; gender, age group, housing (alone or with family members), employment status. They were also asked to provide open ended feedback on the form, limited to 600 characters.

Response scale:

0. I cannot do this
1. I experience severe deterioration
2. I cannot do other activities that day AND the day(s) afterwards
3. I cannot do other activities that day
4. I have to reduce other activities the same day
5. This rarely affects other activity
6. Unproblematic – does not affect other activities

A. Personal hygiene / basic functions

1. Using WC (not bedpan/ bedside commode) (A)
2. Brushing teeth without assistance (X)
3. Showering seated with assistance (A)
4. Showering seated without assistance (A)
5. Showering standing up (A)
6. Getting up and be out if bed for at least an hour (A)
7. Getting dressed (A, N)

B. Walking and other physical activity
8. Walking a short distance indoors, between bedroom and bathroom or kitchen (N)
9. Walking a short continuous distance, approximately 100 m/110 yards (indoors or outdoors) (N)
10. Walking between 100 m/110 yards and 1 km - 3/4 mile on level ground (N)
11. Going for longer walk (more than 1 km - 3/4 mile), mostly level ground (N)
12. Going for a longer walk, more than 1 km, hilly, varied terrain (N)
13. Physical activity with increased heartrate continuously for 15 min (N)
14. Physical activity with increased heartrate, mostly continuously for at least ½ hour (N)

C. Being upright
15. Sitting in bed for at least ½ hour (A)
16. Sitting in an upright chair (dining chair) with feet on floor for at least 10 minutes without increasing discomfort (N)
17. Sitting in an upright chair with feet on floor for several hours (N)
18. Standing for 5 minutes (for example queuing/ cooking) (N)
19. Standing for a long time, 30 minutes or more (N)

D. Activities at home
20. Light housework, f ex dusting/ tidying, at least ½ hour continuously (N)
21. Heavier housework, f ex washing floors/ vacuuming, at least ½ hour continuously (N)
22. Doing laundry (sorting, hanging up to dry) (C)
23. Cooking a simple hot meal/ dinner (N)
24. Fixing a simple sandwich (N)
25. Cooking a complicated meal from scratch (more than an hour of preparation) (N)

E. Communication
26. Saying a few words (A)
27. Having a conversation for at least 5 minutes (A)
28. Having a conversation for more than 30 minutes (A)
29. Writing short messages (N)
30. Participating in a conversation with several people (N)
31. Visiting a friend (C)
32. Getting a visit from a friend (C)
33. Participating in a dinner party/ birthday party/ big family event (C)

F. Activities outside the home
34. Being right outside the home, going to the mailbox or similar (A)
35. Doing necessary errands, like doctor’s appointment (X)
36. Doing pleasant activities outside the home, e.g. visiting a café, shopping, visiting friends (N)
37. Going to the shops to get groceries (N)
38. Being a passenger in a car for at least 15 minutes (N)
39. Using public transport (busses/trains) (N)
40. Participating in organized leisure activities (N)

G. Reactions to light and sound
41. Being in a room with dimmed lighting for ½ hour (T)
42. Being in normal indoor lighting (without sunglasses) for 1 hour (T)
43. Being outdoors in daylight without sunglasses at all (T)
44. Being outdoors in daylight without sunglasses for 2 hours (T)
45. Being in an environment with sound from quiet conversation, few people (T)
46. Being in a noisy environment, (shopping mall, cafe, open plan office) for 1 hour (X)
47. Going to a cinema, concert with loud sound or similar (X)

H: Concentration
48. Reading a short text, like a SMS (N)
49. Reading fiction (N)
50. Reading and understand a nonfiction text (at least one A4 page long) (N)
51. Doing simple mental arithmetic (X)
52. Writing short messages on a smartphone or tablet (A)
53. Using social media to stay in touch with others (A)
54. Watching attentively TV (series, news) 1 hour continuously (N)
55. Focusing on a task for more than 10 minutes continuously (N)
56. Focusing on a task for at least 2 hours continuously (N)
57. Focusing for a full working day (office work) (N)

Feedback

Respondents were also asked to suggest improvements to the form in free text.

Round 4

This Round was in English. Introductory instructions

An introduction gave the reason for asking for help from ME/CFS patients in developing the questionnaire:

ME is a disease which is characterized by:
New onset fatigue that has lasted for at least six months, and that reduces previous activity levels by at least 50 %.
These symptoms are also required to get a diagnosis (Canadian Consensus Criteria):

- Post-Exertional Malaise (PEM) – exacerbated symptoms after exertion, and a prolonged restitution period
- Sleep dysfunction (problems falling asleep, unrefreshing sleep)
- Pain (headaches, muscle and joint pain)
- Cognitive problems (problems with concentration and memory, "brainfog")
- Some (but not necessarily all) of the following symptoms: Nausea, dizziness when standing or sitting, fever or chills, tender lymph nodes, sore throat and flu-like symptoms, problems with digestion etc.

We asked about:
Gender (male, female, prefer not to say)
Year of birth

Year of ME/CFS symptom onset
Year of ME/CFS diagnosis
ME/CFS severity degree (ICC criteria)

They were informed:
"It is best to answer the questionnaire together with a person who is with you regularly".

We did not specify the response period in which they should answer.

A close person can answer on behalf of someone with ME/CFS who is too sick to answer by themselves.

We asked:
"On this page we ask you detailed questions about how various activities affect you.
We are asking about your level of functioning, not about symptoms.
Chose the option that is closest to your experience."

For each domain (A to H) below we asked:
"How would each activity affect you?"

Response scale:

0. I cannot do this
1. I experience serious deterioration
2. I can do nothing else the same day and the day(s) after
3. I can do nothing else the same day
4. I would have to reduce other activity the same day
5. This would rarely affect other activities
6. Unproblematic – does not affect other activity

A. Basic functions/personal hygiene
1. Using WC (not bedpan or bedside commode) (A)
2. Brushing teeth without assistance (X)
3. Showering seated with assistance (A)
4. Showering seated without assistance (A)
5. Showering standing up (A)
6. Getting up and be out if bed for at least an hour (A)
7. Getting dressed (A, N)

B. Walking and other physical activity
8. Walking a short distance indoors, between bedroom and bathroom or kitchen (N)
9. Walking a short continuous distance, approximately 100 m/110 yards (indoors or outdoors) (N)
10. Walking between 100 m/110 yards and 1 km - 3/4 mile on level ground (N)
11. Going for longer walk (more than 1 km - 3/4 mile), mostly level ground (N)
12. Going for a longer walk, more than 1 km, hilly, varied terrain (N)
13. Physical activity with increased heartrate continuously for at least 15 min (N)
14. Physical activity with increased heartrate, mostly continuously for at least ½ hour (N)

C. Being upright
15. Sitting in bed for at least ½ hour (A)
16. Sitting in an upright chair (dining chair) with feet on floor for at least 10 minutes (N)

17. Sitting in an upright chair (dining chair) with feet on floor for several hours (N)
18. Standing for 5 minutes (for example queing/cooking) (N)
19. Standing for a long time, 30 minutes or more (N)

D. Activities in the home
20. Light housework, dusting, tidying, at least ½ hour continuously (N)
21. Heavier housework, f ex washing floors, vacuuming, at least ½ hour continuously (N)
22. Doing laundry (sorting, hanging up to dry, folding) (C)
23. Cooking a simple hot meal (N)
24. Fixing a simple meal, sandwiches/cereal (N)
25. Cooking a complicated meal from scratch (more than an hour of preparation) (N)

E. Communication
26. Saying a few words (A)
27. Having a conversation for at least 5 minutes (A)
28. Having a conversation for more than 30 minutes (A)
29. Writing short messages (N)
30. Participating in a conversation with three or more people for 30 minutes (N)
31. Visiting a friend (C)
32. Getting a visit from a friend (C)
33. Participating in a dinner party, birthday party, big family event or similar (C)

F. Activities outside the home
34. Being right outside the home, going to the mailbox or similar (A)
35. Doing necessary errands, like doctor's appointment (X)
36. Doing pleasant activities outside the home, e.g. visiting a café, shopping, visiting friends (N)
37. Going to the shops to get groceries (N)
38. Being a passenger in a car for at least 15 minutes (N)
39. Using public transport (busses/trains) (N)
40. Participating in organized leisure activities (classes, sports) (N)

G. Reactions to light and sound
41. Being in a room with dimmed lighting for at least ½ hour (T)
42. Being in normal indoor lighting (without sunglasses) for 1 hour (T)
43. Being outdoors in daylight for 2 hours without sunglasses (T)
44. Being in an environment with sound from quiet conversation, few people (T)
45. Being in a noisy environment, (shopping mall, cafe, open plan office) for at least 1 hour (X)
46. Going to a cinema, concert or similar (very loud) (X)

H: Concentration
47. Reading a short text, like a sms (N)
48. Reading fiction (N)
49. Reading and understand a nonfiction text, at least one page long (N)
50. Doing simple mental arithmetic (X)
51. Writing short messages on a smartphone or tablet (A)
52. Using social media to stay in touch with others (A)
53. Watching TV (series, news) (N)
54. Focusing on a task for 10 minutes continuously (N)
55. Focusing on a task for at least 2 hours continuously (N)
56. Focusing for a full working day (office work) (N)

Changes made from Round 4 to Round 5 questionnaire:
Numbers below are based on FUNCAP55. Round 5 FUNCAP55 described first.

A. 7. Getting dressed in regular clothes. "..regular clothes" added.
B. 9. Walking a short continuous distance, approx. 100 m (length of a football field), in- or outdoors. "..(length of a football field)" added.
B. 10. Walking between approx. 100 m and 1 km on level ground (length of 1 to 10 football fields). ".. (length of 1 to 10 football fields)" added.
B. 13. "Physical activity with increased heart rate, for approx. 15 min". "..at least" in Round 4 version was changed to ".. approx.".
B. 14. "..at least." changed to "approx."
C.15, C16, C17. C.19: ".. at least." changed to "approx."
C.18. "Standing for 5 minutes (for example queuing/ cooking)" changed to "Standing up for approx. 5 minutes, e.g. while queuing or while cooking"
D. 20, D.21. D.25: ".. at least." changed to "approx."
E. 27, E.28, E.30: : ".. at least." changed to "approx."
E. 29. "Writing short messages" changed to "Writing a short message by hand".
E. 31. "Socializing with friends for approx. 1 hour" was substituted instead of the two items "Visiting a friend" and "Getting a visit from a friend".
F. 33. "Being right outside the home, going to the mailbox or similar" changed to "Stepping right outside your home".
F. 36. "Doing pleasant activities outside the home, eg. Visiting a café, shopping, visiting friends" changed to "Doing enjoyable leisure activities, such as going to a café, non-essential shopping etc".
F. 37. ".. at least." changed to "approx.".
G. 40. ".. at least." changed to "approx."
G41, G42, G44 ".. for 1 hour" changed to ".. for approx. 1 hour".
H. 47. "Reading fiction" changed to "Reading fiction/light reading".
H. 48. "Reading and understand a non- fiction text, at least one page long" replaced with "Reading and understanding a non-fiction text, such as an official document one A4 page long"

FUNCAP55 items and corresponding ICF WHO functions:

Table S1. FUNCAP55 items and the nearest, corresponding WHO International classification of functioning, disability and health - ICF , function [4]. More detailed description of each function is provided in the WHO ICF manual.

	FUNCAP55 item	ICF WHO function
**A**	**Personal hygiene / basic functions**	
1	Using the toilet (not bedpan or bedside commode)	d5300/01 Regulating urination/ defecation
2	Brushing your teeth without assistance	d5201 Caring for teeth
3	Showering seated, with assistance	d5101 Washing body parts
4	Showering seated, without assistance	d5101 Washing body parts
5	Showering standing up	d5101 Washing body parts
6	Getting up and staying out of bed for approx. 1 hour	None fitting
7	Getting dressed in regular clothes	d5400 Putting on clothes
**B**	**Walking – moving around**	
8	Walking a short distance indoors, from one room to another	d4600 Moving around within the home
9	Walking a short continuous distance, approx. 100 m (length of a football field), in- or outdoors	d4500 Walking short distances (< 1 km)
10	Walking between approx. 100 m and 1 km on level ground (length of 1 to 10 football fields)	d4500 Walking short distances (< 1 km)

	FUNCAP55 item	ICF WHO function
11	Going for a longer walk. Approx. 1 km (0.6 mile), mostly level ground	d4500/01 Walking long distances (>1km)
12	Going for a longer walk. Approx. 1 km (0.6 mile), hilly or varied terrain	d4502 Walking on different surfaces (on sloping, uneven, or moving surfaces)
13	Physical activity with increased heart rate, for approx. 15 min	d4550 General physical endurance
14	Physical activity with increased heart rate, for approx. ½ hour	d4550 General physical endurance
**C**	**Being upright**	
15	Sitting in bed for approx. ½ hour	d4153 Maintaining a sitting position
16	Sitting in an upright chair (dining chair) with feet on floor for approx. 10 minutes	d4153 Maintaining a sitting position
17	Sitting in an upright chair (dining chair) with feet on floor for approx. 2 hours	d4153 Maintaining a sitting position
18	Standing up for approx. 5 minutes, e.g. while queuing or while cooking	d4154 Maintaining a standing position
19	Standing up for a long time – approx. ½ hour	d4154 Maintaining a standing position
**D**	**Activities in the home**	
20	Light housework (dusting, tidying etc.) for approx. 1/2 hour continuously	d6402 Cleaning living area
21	Heavier housework (washing floors, vacuuming etc.) for approx. 1/2 hour continuously	d6402 Cleaning living area
22	Laundry (sorting, hanging up to dry and folding)	d6400 Washing and drying clothes and garments
23	Making a simple cold meal, such as a sandwich or cereal	d6300 Preparing simple meals
24	Cooking a simple hot meal	d6300 Preparing simple meals
25	Cooking a complicated meal from scratch, approx. 1 hour of preparation	d6301 Preparing complex meals
**E**	**Communication**	
26	Speaking a few words	d330 Speaking
27	Having a conversation for approx. 5 minutes	d5303 Conversing with one person
28	Having a conversation for approx. ½ hour	d5303 Conversing with one person
29	Writing a short message by hand	d345 Writing messages
30	Participating in a conversation with three people for approx. 1/2 hour	d3504 Conversing with many people
31	Socializing with friends for approx. 1 hour	d9205 Socializing
32	Participating in a dinner party, party or family event	d9205 Socializing
**F**	**Activities outside your home**	
33	Stepping right outside your home	d4601 Moving around within buildings other than home
34	Going on a necessary errand, such as a doctor's appointment	None fitting
35	Going to a shop for groceries	d6200 Shopping
36	Doing enjoyable leisure activities, such as going to a café, non-essential shopping etc	d9202 Arts and culture
37	Riding as a passenger in a car for approx. 15 minutes	d4701 Using private motorized transportation
38	Using public transport (bus or train)	d4702 Using public motorized transportation
39	Participating in organized leisure activities such as classes, sports etc	d9201 Sports
**G**	**Reactions to light and sound**	
40	Staying in a room with dim lighting for approx. 1/2 hour	b21020 Light sensitivity

	FUNCAP55 item	ICF WHO function
41	Staying in a room with normal lighting, without sunglasses, for approx. 1 hour	b21020 Light sensitivity
42	Staying outdoors in daylight without sunglasses for approx. 2 hours	b21020 Light sensitivity
43	Staying in an environment with the sound of a few people in quiet conversation	b2408 Sensations associated with hearing and vestibular function
44	Staying in a noisy environment, (shopping mall, café or open plan office) for approx. 1 hour	b2408 Sensations associated with hearing and vestibular function
45	Going to a cinema, concert etc. with high noise levels	b2408 Sensations associated with hearing and vestibular function
**H**	**Concentration**	
46	Reading a short text, such as a mobile phone text message	d166 Reading
47	Reading fiction/light reading	d166 Reading
48	Reading and understanding a non-fiction text, such as an official document one A4 page long	d166 Reading
49	Performing simple mental arithmetic	d172 Calculating
50	Writing short messages on a smartphone or tablet	d345 Writing messages
51	Using social media to stay in touch with others	d345 Writing messages
52	Watching TV (series, news)	e5600 Media services
53	Focusing on a task for approx. 10 minutes continuously	d160 Focusing attention
54	Focusing on a task for approx. 2 hours continuously	d160 Focusing attention
55	Managing a full working day (non-physical work such as office work, classes or lectures)	d845/d820 Acquiring, keeping and terminating job/ School education

ICF WHO scoring of functions [4]**:**
Generic qualifier with the negative scale, used to indicate the extent or magnitude of an impairment:

xxx.0 NO impairment	(none, absent, negligible,…)	0-4 %
xxx.1 MILD impairment	(slight, low,…)	5-24 %
xxx.2 MODERATE impairment	(medium, fair,...)	25-49 %
xxx.3 SEVERE impairment	(high, extreme, …)	50-95 %
xxx.4 COMPLETE impairment	(total,…)	96-100 %
xxx.8 not specified		
xxx.9 not applicable		

"The percentages are to be calibrated in different domains with reference to population standards as percentiles. For this quantification to be used in a uniform manner, assessment procedures need to be developed through research."

References

1. Brage S, F.N., Knutsrod OG, Reiso H, Ryen A. Norsk Funksjonsskjema – et nytt instrument ved sykmelding og uførhetsvurdering. Tidsskr Nor Lægeforen 2004, 124, 2472-2474.
2. Nijs, J.e.a. Psychometric Properties of the Dutch Chronic Fatigue Syndrome–Activities and Participation Questionnaire (CFS-APQ). Physical Therapy 2003, 83, 444-454.
3. Sommerfelt, K.; Schei, T.; Angelsen, A. Severe and Very Severe Myalgic Encephalopathy/Chronic Fatigue Syndrome ME/CFS in Norway: Symptom Burden and Access to Care. J Clin Med 2023, 12, doi:10.3390/jcm12041487.
4 World Health Organization (WHO). The International Classification of Functioning, Disability and Health (ICF). 2001. http://www.who.int/classifications/icf/en/
