# Supplementary material for: Assessing Functional Capacity in Myalgic Encephalopathy/Chronic Fatigue Syndrome: A Patient-Informed Questionnaire
Source: J Clin Med. 2024 Jun 14;13(12):3486. doi: 10.3390/jcm13123486 (PMC11204454; doi:10.3390/jcm13123486)
Supplement: Supplementary file 1 [file jcm-13-03486-s001.zip › jcm-2965438 - Supplementary material S2.pdf]

Supplementary Material S2: FUNCAP

Table S2. Correlations among FUNCAP27 A to H sub-scores (and Total Score, i.e. the mean of the eight A to H sub-scores) for the ME/CFS Round 5 and 4 respondents. All Pearson Correlation p-values were < 0.008 (with Bonferroni correction).

Round 5. Norwegian (n=1263):

Domains, ME/CFS respondents	A	B	C	D	E	F	G	H	TS
A. Personal hygiene / basic functions	1	0.77	0.79	0.70	0.69	0.74	0.66	0.61	0.86
B. Walking / moving around		1	0.76	0.77	0.71	0.82	0.66	0.65	0.88
C. Being upright			1	0.74	0.75	0.76	0.71	0.66	0.89
D. Activities in home				1	0.73	0.81	0.66	0.68	0.88
E. Communication					1	0.78	0.73	0.77	0.88
F. Activities outside your home						1	0.73	0.71	0.92
G. Reactions to light and sound							1	0.67	0.84
H. Concentration								1	0.81
TS. Total Score (mean of A-H sub-scores)									1

Round 4. International/ English (n=1387):

Domains, ME/CFS respondents	A	B	C	D	E	F	G	H	TS
A. Personal hygiene / basic functions	1	0.75	0.79	0.65	0.68	0.71	0.66	0.61	0.85
B. Walking / moving around		1	0.75	0.76	0.69	0.81	0.64	0.59	0.87
C. Being upright			1	0.71	0.74	0.76	0.70	0.66	0.89
D. Activities in home				1	0.69	0.83	0.64	0.64	0.87
E. Communication					1	0.77	0.72	0.78	0.87
F. Activities outside your home						1	0.69	0.67	0.91
G. Reactions to light and sound							1	0.66	0.83
H. Concentration								1	0.80
TS. Total Score (mean of A-H sub-scores)									1

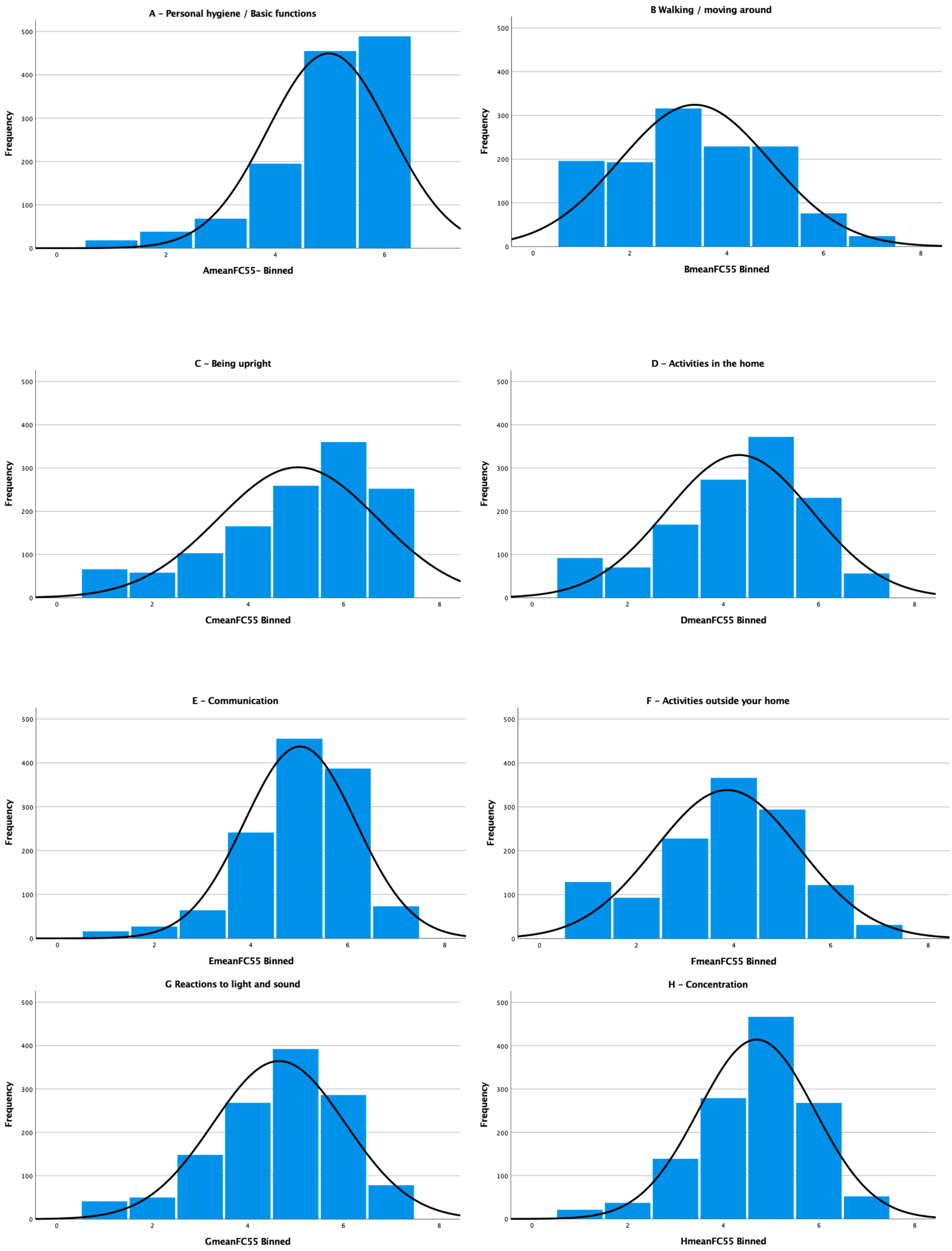

Figure S1. Histograms of the eight A to H domain sub-scores for Round 5 FUNCAP55 ME/CFS respondents (n=1263).

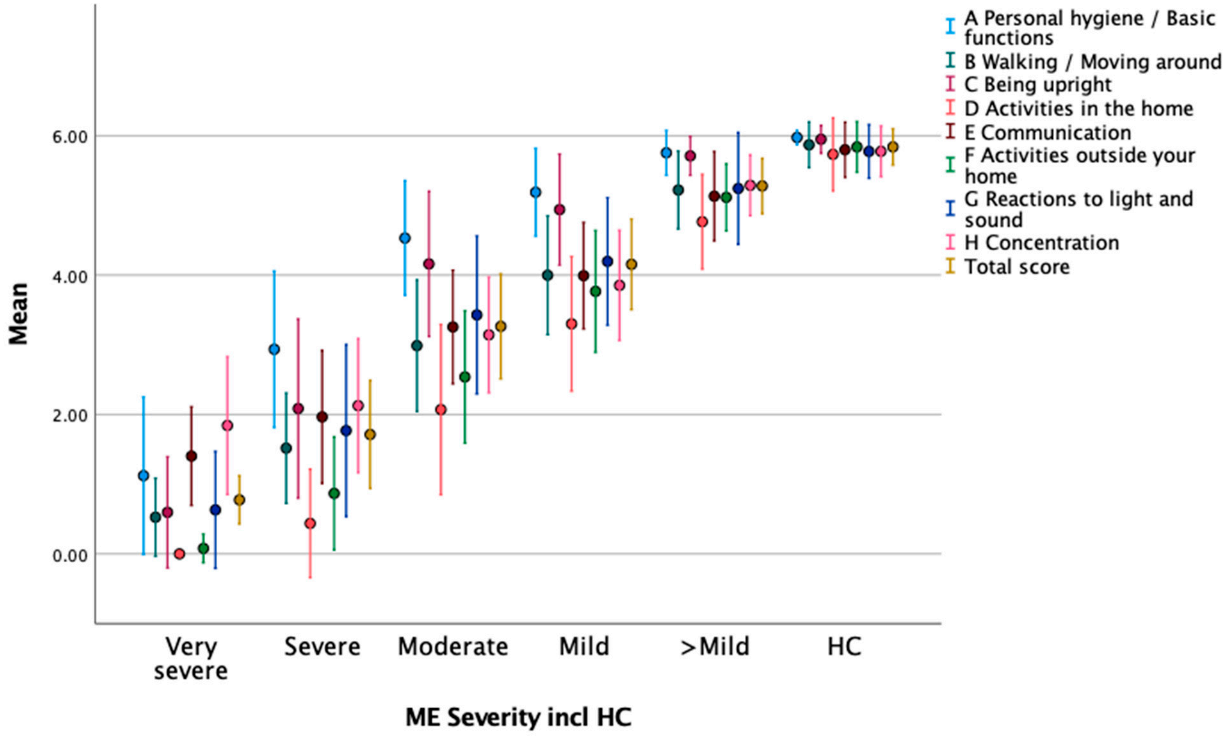

Figure S2. Round 5. Mean (+ - 1 SD) A to H sub-scores and Total Score on the FUNCAP27 questionnaire for the ME/CFS (n=1263) and healthy controls (HC, n=178) respondents according to ME/CFS severity degree which was as follows: *Very severe* (n=19), *severe* (n=136), *moderate* (n=733), *mild* (n=360), *better than mild* (n=15).

Table S3. Round 5 mean (SD) FUNCAP55 A to H domain sub-scores according to ME/CFS severity degrees. HC were not included in statistical analyses. ANOVA analyses for each domain were all statistically significant with $p < 0.001$. Post hoc tests (Tukey) between groups were all statistically significant with $p < 0.001$ (B: *Very severe* vs *severe* p = 0.03. C: *Mild* vs *>mild* p = 0.009. E: *Very severe* vs *severe* p = 0.003) except: A: *Mild* vs *>mild* p = 0.1 and H: *Very severe* vs *severe* p = 0.08. The numbers in each severity were: *Very severe* (19), *severe* (n=136), moderate (n=733), *mild* (n=360) and *better than mild* (n=15).

	Very severe	Severe	Moderate	Mild	>Mild	HC
A. Personal hygiene / basic functions	1.2 (1.0)	2.9 (1.1)	4.6 (0.8)	5.3 (0.6)	5.8 (0.3)	6.0 (0.1)
B. Walking / moving around	0.3 (0.3)	0.8 (0.6)	2.1 (0.9)	3.1 (0.8)	4.2 (0.6)	5.1 (0.3)
C. Being upright	0.5 (0.6)	1.9 (1.2)	4.0 (1.0)	4.8 (0.8)	5.6 (0.3)	5.9 (0.2)
D. Activities in home	0.04 (0.09)	1.3 (1.0)	3.2 (0.9)	4.2 (0.7)	5.3 (0.4)	5.9 (0.3)
E. Communication	2.0 (0.9)	2.6 (1.0)	3.9 (0.7)	4.5 (0.6)	5.4 (0.5)	5.9 (0.3)
F. Activities outside your home	0.1 (0.2)	1.0 (0.8)	2.8 (0.9)	4.0 (0.8)	5.3 (0.4)	5.9 (0.3)
G. Reactions to light and sound	1.1 (0.8)	2.1 (1.0)	3.5 (0.9)	4.2 (0.8)	5.3 (0.6)	5.8 (0.3)
H. Concentration	2.0 (1.1)	2.5 (1.0)	3.6 (0.8)	4.2 (0.7)	5.5 (0.3)	5.8 (0.3)
TS. Total Score (mean of A-H sub-scores)	0.9 (0.4)	1.9 (0.8)	3.5 (0.7)	4.3 (0.6)	5.3 (0.4)	5.8 (0.2)

Table S4. Round 5 mean (SD) FUNCAP27 A to H domain sub-scores according to ME/CFS severity degrees. HC were not included in statistical analyses. ANOVA analyses for each domain were all statistically significant with $p < 0.001$. Post hoc tests (Tukey) between groups were all statistically significant with $p < 0.001$ (C: *Mild* vs *>mild* p = 0.03. E: *Very severe* vs *severe* p = 0.04. F: *Very severe* vs *severe* p = 0.003. G: *Mild* vs *>mild* p = 0.002) except: A: *Mild* vs *>mild* p = 0.06. D: *Very severe* vs *severe* p = 0.48. H: *Very severe* vs *severe* p = 0.63. The numbers in each severity were: Very severe (19), severe (n=136), moderate (n=733), mild (n=360) and better than mild (n=15).

	Very severe	Severe	Moderate	Mild	>Mild	HC
A. Personal hygiene / basic functions	1.1 (1.1)	2.9 (1.1)	4.5 (0.8)	5.2 (0.6)	5.8 (0.3)	6.0 (0.1)
B. Walking / moving around	0.5 (0.6)	1.5 (0.8)	3.0 (0.9)	4.0 (0.9)	5.2 (0.6)	5.1 (0.3)
C. Being upright	0.6 (0.8)	2.1 (1.3)	4.2 (1.0)	4.9 (0.8)	5.7 (0.3)	5.9 (0.2)
D. Activities in home	0.0 (0.0)	0.4 (0.8)	2.1 (1.2)	3.3 (1.9)	4.8 (0.7)	5.7 (0.5)
E. Communication	1.4 (0.7)	2.0 (1.0)	3.3 (0.8)	4.0 (0.8)	5.1 (0.6)	5.8 (0.4)
F. Activities outside your home	0.1 (0.2)	0.9 (0.8)	2.5 (0.9)	3.8 (0.9)	5.1 (0.5)	5.8 (0.4)
G. Reactions to light and sound	0.6 (0.8)	1.8 (1.2)	3.4 (1.1)	4.2 (0.9)	5.2 (0.8)	5.8 (0.4)
H. Concentration	1.8 (1.0)	2.1 (1.0)	3.1 (0.8)	3.9 (0.8)	5.3 (0.4)	5.8 (0.4)
TS. Total Score (mean of A-H sub-scores)	0.8 (0.3)	1.7 (0.8)	3.3 (0.8)	4.2 (0.6)	5.3 (0.4)	5.8 (0.3)

FUNCAP55. Questionnaire on functional capacity

This questionnaire evaluates your functional capacity for a range of activities. No days are the same. Base your response on an average day during the last month – not the worst nor the best. If a question concerns an activity that you have not performed, such as showering while seated because you always shower standing up, then score as you think this activity would have affected you. Items described include necessary activities to perform them. Example: *"Going to a shop for groceries"* includes getting dressed and as necessary travelling.

It is a good idea to answer the questionnaire together with someone who sees you in everyday life.

What are the consequences for you if you perform the activities described below?
To what extent does this affect how much else you can do?

A to H: Scored 0-6:
0: I cannot do this
1: My capacity will be severely reduced for at least three days
2: I can do little else on the same day and for one to two days afterwards
3: I can do little else on the same day
4: I must limit other activities on the same day
5: This rarely affects other activities
6: Unproblematic – does not affect other activities

		0	1	2	3	4	5	6
**A**	**Personal hygiene / basic functions**							
1	Using the toilet (not bedpan or bedside commode)							
2	Brushing your teeth without assistance							
3	Showering seated, with assistance							
4	Showering seated, without assistance							
5	Showering standing up							
6	Getting up and staying out of bed for approx. 1 hour							
7	Getting dressed in regular clothes							
**B**	**Walking – moving around**							
8	Walking a short distance indoors, from one room to another							
9	Walking a short continuous distance, approx. 100 m (length of a football field), in- or outdoors							
10	Walking between approx. 100 m and 1 km on level ground (length of 1 to 10 football fields)							
11	Going for a longer walk. Approx. 1 km (0.6 mile), mostly level ground							
12	Going for a longer walk. Approx. 1 km (0.6 mile), hilly or varied terrain							
13	Physical activity with increased heart rate, for approx. 15 min							
14	Physical activity with increased heart rate, for approx. ½ hour							
**C**	**Being upright**							
15	Sitting in bed for approx. ½ hour							
16	Sitting in an upright chair (dining chair) with feet on floor for approx. 10 minutes							
17	Sitting in an upright chair (dining chair) with feet on floor for approx. 2 hours							
18	Standing up for approx. 5 minutes, e.g. while queuing or while cooking							
19	Standing up for a long time – approx. ½ hour							
**D**	**Activities in the home**							
20	Light housework (dusting, tidying etc.) for approx. 1/2 hour continuously							

		0	1	2	3	4	5	6
21	Heavier housework (washing floors, vacuuming etc.) for approx. 1/2 hour continuously							
22	Laundry (sorting, hanging up to dry and folding)							
23	Making a simple cold meal, such as a sandwich or cereal							
24	Cooking a simple hot meal							
25	Cooking a complicated meal from scratch, approx. 1 hour of preparation							
**E**	**Communication**							
26	Speaking a few words							
27	Having a conversation for approx. 5 minutes							
28	Having a conversation for approx. ½ hour							
29	Writing a short message by hand							
30	Participating in a conversation with three people for approx. 1/2 hour							
31	Socializing with friends for approx. 1 hour							
32	Participating in a dinner party, party or family event							
**F**	**Activities outside your home**							
33	Stepping right outside your home							
34	Going on a necessary errand, such as a doctor's appointment							
35	Going to a shop for groceries							
36	Doing enjoyable leisure activities, such as going to a café, non-essential shopping etc							
37	Riding as a passenger in a car for approx. 15 minutes							
38	Using public transport (bus or train)							
39	Participating in organized leisure activities such as classes, sports etc							
**G**	**Reactions to light and sound**							
40	Staying in a room with dim lighting for approx. 1/2 hour							
41	Staying in a room with normal lighting, without sunglasses, for approx. 1 hour							
42	Staying outdoors in daylight without sunglasses for approx. 2 hours							
43	Staying in an environment with the sound of a few people in quiet conversation							
44	Staying in a noisy environment, (shopping mall, café or open plan office) for approx. 1 hour							
45	Going to a cinema, concert etc. with high noise levels							
**H**	**Concentration**							
46	Reading a short text, such as a mobile phone text message							
47	Reading fiction/light reading							
48	Reading and understanding a non-fiction text, such as an official document one A4 page long							
49	Performing simple mental arithmetic							
50	Writing short messages on a smartphone or tablet							
51	Using social media to stay in touch with others							
52	Watching TV (series, news)							
53	Focusing on a task for approx. 10 minutes continuously							
54	Focusing on a task for approx. 2 hours continuously							
55	Managing a full working day (non-physical work such as office work, classes or lectures)							

FUNCAP27. Questionnaire on functional capacity

This questionnaire evaluates your functional capacity for a range of activities. No days are the same. Base your response on an average day during the last month – not the worst nor the best. If a question concerns an activity that you have not performed, such as showering while seated because you always shower standing up, then score as you think this activity would have affected you.
Items described include necessary activities to perform them. Example: *"Going to a shop for groceries"* includes getting dressed and as necessary travelling.

It is a good idea to answer the questionnaire together with someone who sees you in everyday life.

What are the consequences for you if you perform the activities described below?
To what extent does this affect how much else you can do?

A to H: Scored 0-6:
0: I cannot do this
1: My capacity will be severely reduced for at least three days
2: I can do little else on the same day and for one to two days afterwards
3: I can do little else on the same day
4: I must limit other activities on the same day
5: This rarely affects other activities
6: Unproblematic – does not affect other activities

		0	1	2	3	4	5	6
**A**	**Personal hygiene / basic functions**							
1	Using the toilet (not bedpan or bedside commode)							
2	Showering standing up							
3	Getting dressed in regular clothes							
**B**	**Walking – moving around**							
4	Walking a short distance indoors, from one room to another							
5	Walking between approx. 100 m and 1 km on level ground (length of 1 to 10 football fields)							
6	Physical activity with increased heart rate, for approx. 15 min							
**C**	**Being upright**							
7	Sitting in bed for approx. ½ hour							
8	Sitting in an upright chair (dining chair) with feet on floor for approx. 2 hours							
9	Standing up for approx. 5 minutes, e.g. while queuing or while cooking							
**D**	**Activities in the home**							
10	Heavier housework (washing floors, vacuuming etc.) for approx. 1/2 hour continuously							
11	Cooking a complicated meal from scratch, approx. 1 hour of preparation							
**E**	**Communication**							
12	Having a conversation for approx. 5 minutes							
13	Participating in a conversation with three people for approx. 1/2 hour							
14	Participating in a dinner party, party or family event							
**F**	**Activities outside your home**							
15	Stepping right outside your home							
16	Going to a shop for groceries							
17	Using public transport (bus or train)							
18	Participating in organized leisure activities such as classes, sports etc.							

		0	1	2	3	4	5	6
G	**Reactions to light and sound**							
19	Staying in a room with normal lighting, without sunglasses, for approx. 1 hour							
20	Staying outdoors in daylight without sunglasses for approx. 2 hours							
21	Staying in a noisy environment, (shopping mall, café or open plan office) for approx. 1 hour							
H	**Concentration**							
22	Reading a short text, such as a mobile phone text message							
23	Reading and understanding a non-fiction text, such as an official document one A4 page long							
24	Using social media to stay in touch with others							
25	Focusing on a task for approx. 10 minutes continuously							
26	Focusing on a task for approx. 2 hours continuously							
27	Managing a full working day (non-physical work such as office work, classes or lectures)							

Norwegian:
FUNCAP55. Spørreskjema om funksjonskapasitet

Dette spørreskjemaet har spørsmål som kartlegger din funksjonskapasitet innenfor ulike felt. Ingen dager er like. Vi ber deg ta utgangspunkt i en "middels" dag den siste måneden- ikke den verste eller den beste. Hvis det er en aktivitet du ikke har gjort, f.eks. å dusje sittende fordi du dusjer stående, skår slik du tror det ville påvirke deg. Spørsmålene inkluderer nødvendige aktiviteter for å utføre dem. For eksempel inkluderer «*Reise til en butikk og handle dagligvarer*» å kle på seg og nødvendig reise.

Det er en god ide å svare sammen med en som kjenner til hvordan du har det i hverdagen.

Hva er konsekvensene for deg av aktivitetene beskrevet under?
I hvilken grad påvirker det hvor mye annet du kan gjøre?
A til H skåres 0-6:

0: Jeg kan IKKE gjøre dette
1: Jeg får kraftig nedsatt kapasitet i minst tre dager
2: Jeg kan gjøre lite annet samme dag og i en, eventuelt to dager etter
3: Jeg kan gjøre lite annet samme dag
4: Jeg må redusere annen aktivitet samme dag
5: Det påvirker sjelden annen aktivitet
6: Uproblematisk - påvirker ikke annen aktivitet

		0	1	2	3	4	5	6
**A**	**Personlig hygiene/ basis-funksjoner**							
1	Bruke WC (ikke bekken eller dostol)							
2	Pusse tenner uten hjelp							
3	Dusje sittende med hjelp							
4	Dusje sittende uten hjelp							
5	Dusje stående							
6	Stå opp og være ute av sengen i ca. 1 time							
7	Kle på deg vanlige klær							
**B**	**Gå - bevege seg**							
8	Gå en liten strekning inne, fra et rom til et annet							
9	Gå en kort sammenhengende distanse, ca. 100 meter (lengden av en fotballbane). Ute eller inne							
10	Gå ca. 100 m til 1 km på flat mark (lengden av 1 – 10 fotballbaner)							
11	Gå en lengere tur. Ca. 1 km, mest flatt							
12	Gå en lengere tur. Ca. 1 km, i bakker eller ulendt terreng							
13	Fysisk aktivitet som gir høy puls, tilnærmet kontinuerlig i ca. 15 minutter							
14	Trening med høy puls, tilnærmet kontinuerlig i ca. 1/2 time							
**C**	**Være oppreist**							
15	Sitte i sengen i ca. 1/2 time							
16	Sitte på vanlig stol (spisestol) med bena på gulvet i ca. 10 minutter							
17	Sitte på vanlig stol (spisestol) med bena på gulvet i ca. 2 timer							
18	Stå i ca. 5 minutter, f.eks. i kø, eller mens du lager mat							
19	Stå lenge - i ca. ½ time							
**D**	**Aktiviteter i hjemmet**							
20	Lett husarbeid som å rydde, tørke støv eller tilsvarende i ca. 1/2 time kontinuerlig							

		0	1	2	3	4	5	6
21	Tyngre husarbeid, som å vaske gulv, støvsuge eller tilsvarende i ca. 1/2 time kontinuerlig							
22	Vaske klær (sortere, henge opp og brette)							
23	Lage et enkelt kaldt måltid. Som brødskive eller kornblanding							
24	Lage et enkelt varmt måltid							
25	Lage et komplisert måltid fra bunnen av som tar ca. 1 time å lage							
**E**	**Kommunikasjon**							
26	Si noen få ord							
27	Ha en samtale på ca. 5 minutter							
28	Ha en samtale på ca. ½ time							
29	Skrive korte meldinger for hånd							
30	Delta i en samtale med tre personer i ca. 1/2 time							
31	Være med venner i ca. 1 time							
32	Delta i middagsselskap, fest eller familiebegivenhet							
**F**	**Aktiviteter utenfor hjemmet**							
33	Gå rett utenfor hjemmet							
34	Gjøre nødvendige ærender, f.eks. legetime							
35	Reise til en butikk og handle dagligvarer							
36	Gjøre lystbetont aktivitet som kafebesøk, ikke-nødvendig handling, osv							
37	Være passasjer i bil i ca. 15 minutter							
38	Bruke kollektivtransport (buss eller tog)							
39	Drive med organiserte fritidsaktiviteter som kurs eller sport, osv							
**G**	**Reaksjon på lyd og lys**							
40	Oppholde deg i dempet belysning i ca. ½ time							
41	Oppholde deg i normal inne-belysning uten solbriller i ca. 1 time							
42	Være ute i dagslys uten solbriller i ca. 2 timer							
43	Være i omgivelser med lyd fra rolig samtale med få personer							
44	Være i støyende omgivelser (kjøpesenter, kafé eller kontorlandskap) i 1 ca. time							
45	Være på kino, konsert eller liknende med høyt lydnivå							
**H**	**Konsentrasjon**							
46	Lese en kort tekst, f.eks. en tekstmelding på mobiltelefon							
47	Lese skjønnlitteratur eller underholdningslitteratur							
48	Lese og forstå en fagtekst, som f.eks. et offentlig dokument på en A4 side							
49	Gjøre enkel hoderegning							
50	Skrive korte meldinger på mobil eller nettbrett							
51	Bruke sosiale media for å holde kontakt med andre							
52	Se på TV (serier, nyheter)							
53	Konsentrere deg om en oppgave i ca. 10 minutter sammenhengende							
54	Konsentrere deg om en oppgave i ca. 2 timer sammenhengende							
55	Gjennomføre en hel stillesittende dag (f.eks. kontorjobb, skoledag eller studier)							

Norwegian:
FUNCAP27. Spørreskjema om funksjonskapasitet

Dette spørreskjemaet har spørsmål som kartlegger din funksjonskapasitet innenfor ulike felt. Ingen dager er like. Vi ber deg ta utgangspunkt i en "middels" dag den siste måneden- ikke den verste eller den beste. Hvis det er en aktivitet du ikke har gjort, f.eks. å dusje sittende fordi du dusjer stående, skår slik du tror det ville påvirke deg. Spørsmålene inkluderer nødvendige aktiviteter for å utføre dem. For eksempel inkluderer «Reise til en butikk og handle dagligvarer» å kle på seg og nødvendig reise.

Det er en god ide å svare sammen med en som kjenner til hvordan du har det i hverdagen.

Hva er konsekvensene for deg av aktivitetene beskrevet under?
I hvilken grad påvirker det hvor mye annet du kan gjøre?
A til H skåres 0-6:

0: Jeg kan IKKE gjøre dette
1: Jeg får kraftig nedsatt kapasitet i minst tre dager
2: Jeg kan gjøre lite annet samme dag og i en, eventuelt to dager etter
3: Jeg kan gjøre lite annet samme dag
4: Jeg må redusere annen aktivitet samme dag
5: Det påvirker sjelden annen aktivitet
6: Uproblematisk - påvirker ikke annen aktivitet

		0	1	2	3	4	5	6
**A**	**Personlig hygiene/ basis-funksjoner**							
1	Bruke WC (ikke bekken eller dostol)							
2	Dusje stående							
3	Kle på deg vanlige klær							
**B**	**Gå - bevege seg**							
4	Gå en liten strekning inne, fra et rom til et annet							
5	Gå ca. 100 m til 1 km på flat mark (lengden av 1 – 10 fotballbaner)							
6	Fysisk aktivitet som gir høy puls, tilnærmet kontinuerlig i ca. 15 minutter							
**C**	**Være oppreist**							
7	Sitte i sengen i ca. 1/2 time							
8	Sitte på vanlig stol (spisestol) med bena på gulvet i ca. 2 timer							
9	Stå i ca. 5 minutter, f.eks. i kø, eller mens du lager mat							
**D**	Aktiviteter i hjemmet							
10	Tyngre husarbeid, som å vaske gulv, støvsuge eller tilsvarende i ca. 1/2 time kontinuerlig							
11	Lage et komplisert måltid fra bunnen av som tar ca. 1 time å lage							
**E**	Kommunikasjon							
12	Ha en samtale på ca. 5 minutter							
13	Delta i en samtale med tre personer i ca. 1/2 time							
14	Delta i middagsselskap, fest eller familiebegivenhet							
**F**	**Aktiviteter utenfor hjemmet**							
15	Gå rett utenfor hjemmet							
16	Reise til en butikk og handle dagligvarer							
17	Bruke kollektivtransport (buss eller tog)							
18	Drive med organiserte fritidsaktiviteter som kurs eller sport osv.							
**G**	**Reaksjon på lyd og lys**							
19	Oppholde deg i normal inne-belysning uten solbriller i ca. 1 time							

		0	1	2	3	4	5	6
20	Være ute i dagslys uten solbriller i ca. 2 timer							
21	Være i støyende omgivelser (kjøpesenter, kafé eller kontorlandskap) i 1 ca. time							
**H**	**Konsentrasjon**							
22	Lese en kort tekst, f.eks. en tekstmelding på mobiltelefon							
23	Lese og forstå en fagtekst, som f.eks. et offentlig dokument på en A4 side							
24	Bruke sosiale media for å holde kontakt med andre							
25	Konsentrere deg om en oppgave i ca. 10 minutter sammenhengende							
26	Konsentrere deg om en oppgave i ca. 2 timer sammenhengende							
27	Gjennomføre en hel stillesittende dag (f.eks. kontorjobb, skoledag eller studier)							
